# Frequency-Dependent Properties of the Hyperpolarization-Activated Cation Current, I_f_, in Adult Mouse Heart Primary Pacemaker Myocytes

**DOI:** 10.3390/ijms23084299

**Published:** 2022-04-13

**Authors:** Wei Hu, Robert B. Clark, Wayne R. Giles, Colleen Kondo, Henggui Zhang

**Affiliations:** 1 Biological Physics Group, Department of Physics and Astronomy, The University of Manchester, Manchester M13 9PL, UK; wei.hu-3@postgrad.manchester.ac.uk; 2 Department of Physiology and Pharmacology, Cumming School of Medicine, University of Calgary, Calgary, AB T2N 4N1, Canada; rclar@ucalgary.ca (R.B.C.); wgiles@ucalgary.ca (W.R.G.); colleenkondo@gmail.com (C.K.); 3 Key Laboratory of Medical Electrophysiology of Ministry of Education and Medical Electrophysiological Key Laboratory of Sichuan Province, Institute of Cardiovascular Research, Southwest Medical University, Luzhou 646099, China

**Keywords:** mouse heart, sino-atrial node, SAN, spontaneous pacemaker activity, pacemaker depolarization, hyperpolarization-activated current, I_f_, residual activation, mathematical modeling, β adrenergic stimulation

## Abstract

A number of distinct electrophysiological mechanisms that modulate the myogenic spontaneous pacemaker activity in the sinoatrial node (SAN) of the mammalian heart have been investigated extensively. There is agreement that several (3 or 4) different transmembrane ionic current changes (referred to as the voltage clock) are involved; and that the resulting net current interacts with direct and indirect effects of changes in intracellular Ca^2+^ (the calcium clock). However, significant uncertainties, and important knowledge gaps, remain concerning the functional roles in SAN spontaneous pacing of many of the individual ion channel- or exchanger-mediated transmembrane current changes. We report results from patch clamp studies and mathematical modeling of the hyperpolarization-activated current, I_f_, in the generation/modulation of the diastolic depolarization, or pacemaker potential, produced by individual myocytes that were enzymatically isolated from the adult mouse sinoatrial node (SAN). Amphotericin-mediated patch microelectrode recordings at 35 °C were made under control conditions and in the presence of 5 or 10 nM isoproterenol (ISO). These sets of results were complemented and integrated with mathematical modeling of the current changes that take place in the range of membrane potentials (−70 to −50 mV), which corresponds to the ‘pacemaker depolarization’ in the adult mouse SAN. Our results reveal a very small, but functionally important, approximately steady-state or time-independent current generated by residual activation of I_f_ channels that are expressed in these pacemaker myocytes. Recordings of the pacemaker depolarization and action potential, combined with measurements of changes in I_f_, and the well-known increases in the L-type Ca^2+^ current, I_CaL_, demonstrated that I_CaL_ activation, is essential for myogenic pacing. Moreover, after being enhanced (approximately 3-fold) by 5 or 10 nM ISO, I_CaL_ contributes significantly to the positive chronotropic effect. Our mathematical model has been developed in an attempt to better understand the underlying mechanisms for the pacemaker depolarization and action potential in adult mouse SAN myocytes. After being updated with our new experimental data describing I_f_, our simulations reveal a novel functional component of I_f_ in adult mouse SAN. Computational work carried out with this model also confirms that in the presence of ISO the residual activation of I_f_ and opening of I_CaL_ channels combine to generate a net current change during the slow diastolic depolarization phase that is essential for the observed accelerated pacemaking rate of these SAN myocytes.

## 1. Introduction

The electrophysiological and biophysical mechanisms that are responsible for the myogenic, spontaneous firing, or pacemaker activity, in the mammalian heart have been extensively investigated using combinations of conventional microelectrode and patch clamp recordings, as well as pharmacological and molecular methods (for a classical review see [1,2,3,4,5]). There is general agreement that a distinct, small region located within the anatomical sinoatrial node (SAN) functions as the leading or primary pacemaker site [2,6,7,8]. It is also known that interactions among a number of different transmembrane ionic currents (the voltage clock), acting in conjunction with direct and indirect effects of phasic release of Ca^2+^ from intracellular stores (the Ca^2+^ clock) results in a very small, but functionally important, net inward current in the mammalian SAN [9,10,11]. Changes in this net current are responsible for initiating and regulating the slow diastolic depolarization, or pacemaker potential, in spontaneously firing healthy mammalian SAN myocytes. This principle applies at baseline or under control conditions, in the setting of autonomic nervous stimulation, as well as in a number of acute or chronic diseases [11,12,13].

The present study focused on the ‘voltage clock’ components of this primary pacemaker mechanism in the adult mouse heart c.f. [9,10,14]. In particular, we have studied the hyperpolarization-activated, nonselective cation current that has been denoted I_f_, [15,16,17]. This slowly changing time- and voltage-dependent current has sometimes been referred to as ‘the pacemaker current’. However, it is now recognized that alterations in I_f_ represent only one of the essential current changes that combine to regulate the net current profile which drives spontaneous pacemaker activity in the SAN, both under baseline conditions or in response to regulation by the autonomic nervous system. When this ‘autonomic tone’ is significant, the finely balanced current changes resulting from activity of the sympathetic (adrenergic) or parasympathetic (cholinergic) innervation produce classical chronotropic responses [2,18,19,20].

Many of the most informative experimental investigations into the electrophysiological and biophysical basis for SAN pacemaker activity have utilized isolated tissue or single SAN myocyte recordings from the adult mouse heart [20,21,22,23]. In the mouse SAN, and in analogous studies carried out using rat or rabbit SAN preparations, current changes due to I_f_ can be consistently recorded [11,13]. In SAN myocytes, it is well known that I_f_ can be modulated by a number of different factors. These include autonomic transmitters and endogenous natriuretic peptides [20], as well as cardiovascular stimulation such as endurance exercise (for review see [24,25]) and circadian clock activities [26]. Nonetheless, an unequivocal demonstration of the functional role of the very small and also quite slow changes in I_f_ as a ‘pacemaker current’ continues to present a significant technical challenge c.f. [16]. At present some uncertainty remains concerning essential characteristic of I_f_. Examples include:

- Its steady-state voltage dependence.
- Its kinetics or dynamics within the small range of voltages (−65 to −45 mV) that are in the slow diastolic depolarization phase of SAN primary pacemaker activity.
- The sensitivity and size of the changes in I_f_ due to alterations in sympathetic nerve activity, compared to changes in the L-type calcium current, I_CaL_, and/or the predominant K^+^ current, a delayed rectifier K^+^ conductance, I_Kr_.

Accordingly for this study, our three goals were:

- To make high resolution recordings of I_f_ at physiological temperatures in single spontaneously active murine SAN myocytes. In particular, to generate data sets including records of spontaneous pacing and action potentials using a single myocyte patch clamp configuration (amphotericin-mediated whole cell recordings) under conditions that minimize disruption of the intracellular milieu and therefore allow direct comparisons with previously published data sets and biomarkers [23,27].
- To integrate this information into our mathematical model of SAN myocyte primary pacemaker activity [8] and to then use this computational platform to illustrate the relative contributions of I_f_, I_CaL_ and I_Kr_ to the generation of the pacemaker potential c.f. [8,13,23].
- To make experimental recordings and mathematical simulations of the current changes resulting from very low (10 nM), or just threshold, effects of the ß adrenergic agonist, isoproterenol, on both I_f_ and I_CaL_ in the same spontaneously active myocyte in an attempt to further understand the ionic mechanism for the positive chronotropic effects of sympathetic nerve stimulation [20,28,29].

Our experimental results confirm that previously published enzymatic isolation procedures [22,23] can consistently yield the small populations of viable, spontaneously firing and isoproterenol-sensitive individual SAN myocytes that are essential for this type of study. The resulting data sets and analyses provide new information concerning biophysical properties of I_f_, including steady-state activation, kinetics of activation and deactivation and relative sensitivity to ionic and organic inhibitors such as CsCl, BaCl_2_ and zatebradine [30,31,32,33,34]. 

In addition, our amphotericin-mediated patch microelectrode recordings of I_CaL_, at baseline and after bath application of 1–10 nM ISO provide insights concerning the functional role of this Ca^2+^ current in adult mouse SAN pacemaker activity [35,36,37]. Based on this informative (but also somewhat limited) experimental data, an updated mathematical model (see [8,38]) of primary pacemaker activity in the SAN yields new insights into the functional roles of I_f_ and I_CaL_ at baseline and during sympathetic stimulation. 

When this manuscript was being written, two interesting and informative papers regarding the functional roles of I_f_ in the generation of pacemaker activity in the mouse SAN were published [39,40]. Details of these findings will be presented and put into the context of our results in Section 4.

## 2. Results

### 2.1. Physiological Insights

**Figure 1:**
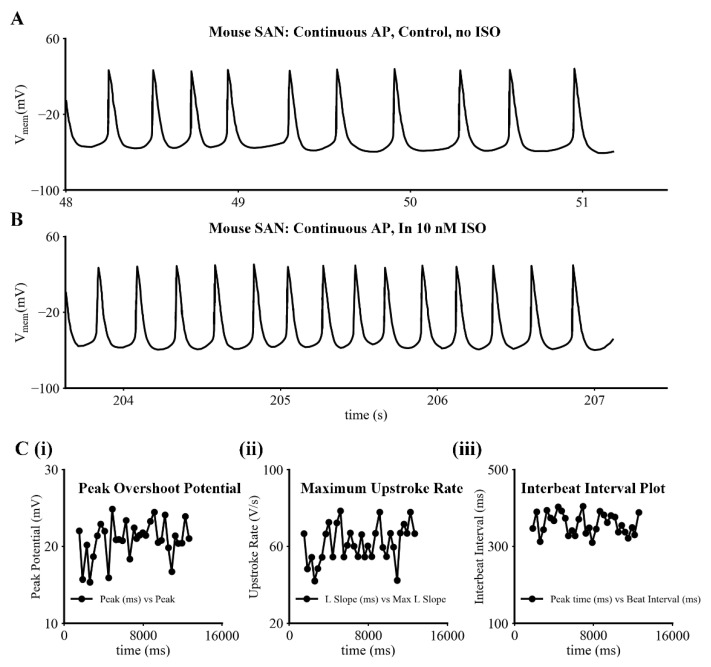
Intracellular recordings of spontaneous pacemaker activity from an adult mouse SAN myocyte under control conditions and in 10 nM Isoproterenol (ISO) at 35 °C. (**A**) Recording of action potentials from primary pacemaker cells under control or baseline conditions. (**B**) Recording of action potentials from the same myocyte as in (**A**) 4 to 5 min after 10 nM ISO was added to the superfusate. (**C**) Numerical values for key parameters (overshoot (**i**), dV/dt (**ii**), interbeat interval (**iii**)) of action potentials provide an indication of the experimental variability in these amphotericin-mediated patch electrode recordings in this study. The measured mean value of the interbeat interval is about 359.3 ms (167 beats/min) with SD = 27.3 ms and SE = 4.9 ms (*n* = 33).

As indicated, our primary objective was to gain additional semiquantitative information concerning the electrophysiological and biophysical properties of I_f_ under conditions that closely approximated a physiological milieu. The data sets in Figure 1 provide representative examples of the SAN pacemaker activity and action potentials that were consistently recorded under control conditions, or at baseline, (Figure 1A) and then subsequently (in the same myocyte) in the presence of 10 nM ISO in the superfusing Tyrode’s solution, (Figure 1B). This physiological level of β adrenergic stimulation by ISO produced an approximately 2.5-fold increase in the spontaneous firing rate. The three sets of reduced data in Figure 1C are included to provide an indication of the variations in key descriptors, including: peak overshoot potential, maximum upstroke rate, and interbeat interval; and also, to allow comparison of our results to comprehensive data sets that have been published previously c.f. [27,39,40]. In addition to the features of the action potentials shown in this figure, it is apparent from the results in Figure 1A,B that the maximum diastolic potential in mouse SAN myocytes that are typical of those that we have recorded from lies between −55 and −60 mV.

**Figure 2:**
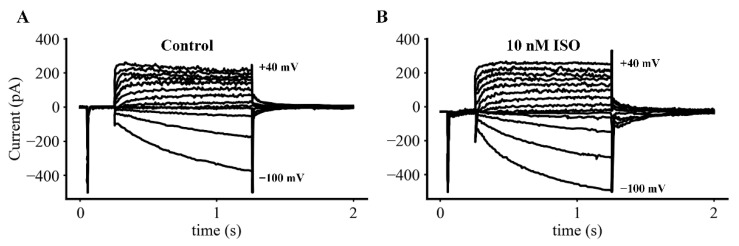
Families of whole cell or macroscopic transmembrane currents in an SAN primary pacemaker myocyte at 35 °C. (**A**) Transmembrane current recording in control conditions. (**B**) Transmembrane current recording in 10 nM ISO. The transmembrane current records in both (**A**,**B**) were obtained in normal Tyrodes in response to 1 s rectangular voltage clamp steps, that displaced the membrane potential from −100 to +40 mV from a holding potential (HP) of −60 mV. These were applied every 8 s in 10 mV increments. A 50 ms pre-pulse to −40 mV was included in all cases to inactivate the transient inward Na^+^ and activate Ca^2+^ currents. This protocol resulted in most of the time- and voltage-dependent outward currents being due to a delayed rectifier K^+^ conductance, I_Kr_; and the slow development of inward currents being generated mainly by I_f_. The superimposed family of currents in (**B**) provide a qualitative indication of the changes in these families of currents recorded 5 min after 10 nM ISO was added to the superfusate. All recordings were made in the amphotericin-mediated whole cell patch clamp configuration (see Methods).

The superimposed families of transmembrane ionic current records shown in both the left and the right panels of Figure 2 illustrate representative data obtained during patch clamp experiments that were carried out to directly compare and contrast important features of the time- and voltage-dependent K^+^ current, I_Kr_, versus those of I_f_ – the hyperpolarization-activated cation current that is the focus of this study. In both panels of this figure, each of the 13 superimposed transmembrane current records were generated in response to 1 s de- or hyperpolarizing rectangular voltage clamp steps applied from a holding potential of −60 mV to membrane potentials in the range of −100 to +40 mV. Each of these voltage clamp commands was preceded by a 50 ms prepulse to −40 mV. This prepulse was applied to inactivate the Na^+^ current. However, as shown in the figure, it also activated L-type Ca^2+^ channels. This observation provided necessary assurance that each preparation that was included in this study generated robust I_CaL_, I_Kr_ and I_f_ records. Comparison of the superimposed families of currents shown in the left panel with those in the right panel reveal significant changes in I_f_ (both in time course and amplitude) following application of 10 nM ISO, as well as much smaller changes in I_Kr_. Note that the peak size of the inward L-type Ca^2+^ current, I_CaL_, is truncated in Figure 2A,B.

**Figure 3:**
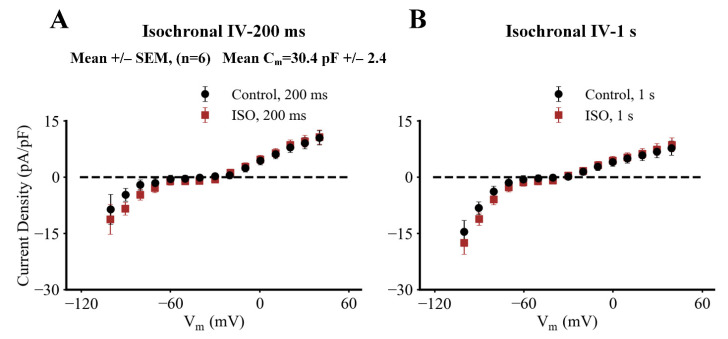
Isochronal current-voltage (I–V) relations corresponding to the families of currents shown in Figure 2. (**A**) The two current-voltage (I–V) relationships were obtained by measuring the maximum outward or inward currents at the end of each 200 ms rectangular voltage clamp step. (**B**) The two superimposed I–V curves are analogous to those on the left except that each data point was obtained following a 1 s rectangular voltage step. Each data set is expressed in terms of current density based on the mean capacitance of each pacemaker myocyte in this series measuring 30.4 pF +/− 2.4, (*n* = 6). Note that in response to the 200 ms clamp steps ISO at 10 nM (red data points) may produce a small increase in inward current in the membrane potential range between approximately −20 to −50 mV; and that this ISO-induced inward current is more clearly observed at strongly hyperpolarized membrane potentials between −80 and −100 mV. The two superimposed I–V curves in the right panel also provide an indication that relatively large and long (1 s) depolarizations or hyperpolarizations can increase transmembrane current in the presence of 10 nM ISO.

Initial insights into plausible physiological roles for I_f_ in SAN pacemaker activity can be obtained by constructing current voltage (I–V) plots based on the raw data from experiments like the one shown in Figure 2. Figure 3 consists of two such I-V plots both of which compare and contrast data obtained at baseline (black circles) with results recorded approximately five minutes after bath application of 10 nM ISO (red squares). The main difference in these two isochronal I–V relations is that the data in the left panel depicts measurements taken at the end of 200 ms voltage clamp steps to each of the membrane potentials shown; while the data in the right hand column was obtained after 1 s steps to these same membrane potentials. In this experiment, the holding potential was −45 mV. The data in both panels illustrate the average of results from six separate experiments. 

These results (and those in Figures S1D and S2B) can be used for inspection of the regions, or voltage ranges, on these I–V curves (between approximately −60 to −40 mV) that are most relevant for illustrating the transmembrane current changes, which generate the pacemaker potential. These findings suggest that I_f_ may produce a small inward current within this voltage range; and also that ß adrenergic stimulation with 10 nM ISO may enhance this small inward current. At more hyperpolarized potentials, e.g., −70 to −100 mV the ability of ISO to enhance I_f_ and to result in faster I_f_ activation kinetics is more apparent. These effects have been reported previously in other SAN preparations [15,16,17,39]; our preliminary analysis did not convincingly demonstrate these small changes (see Section 4). 

The next set of voltage clamp experiments was carried out to reveal important details of I_f_ during very early stages of its activation, and also to confirm the selectivity profile or reversal potential value for I_f_. Previous findings have consistently shown that when I_f_ is first activated there is a significant and very rapid or quasi instantaneous current change, and that this is then followed by the characteristic and very prominent time- and voltage-dependent activation of I_f_ at hyperpolarized membrane potentials. Under physiological conditions it has been reported that the reversal potential for I_f_ is approximately −20 mV; and detailed analyses of I_f_ channel ion selectivity have been interpreted in terms of significant contributions of Na^+^, K^+^ and Ca^2+^ [15,39] as I_f_ current carriers. 

**Figure 4:**
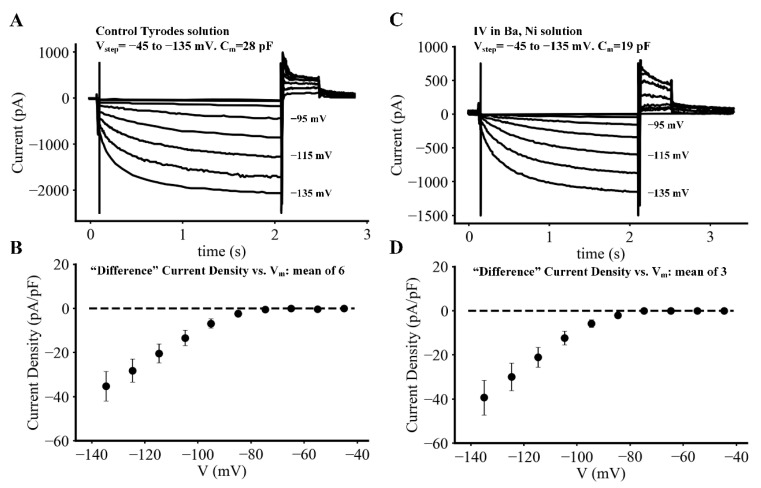
General properties and reversal potential measurements for the hyperpolarization-activated current, I_f_, in adult mouse SAN pacemaker myocytes. (**A**,**B**) Transmembrane current records and I–V in control Tyrodes solution. (**C**,**D**) Transmembrane current records and I-V in Ba, Ni solution. In these experiments, a paired-pulse clamp protocol (P1–P2) was used. In P1, each myocyte was held at −45 mV and progressively larger 2 s hyperpolarizing voltage clamp waveforms were applied in 10 mV increments in the voltage range −55 mV to −135 mV. As shown in (**A**,**B**), most current waveforms consisted of an instantaneous inward current followed, by time- and voltage-dependent development of inward current that increased in size and activated faster with progressive hyperpolarizations. To obtain conditions under which the current change was dominated by I_f_, these measurements were repeated in the presence of BaCl_2_ (0.1 mM) (**C**,**D**) to block the inwardly rectifying background K^+^ current, I_K1_; and NiCl_2_ (0.5 mM) to markedly reduce transmembrane currents generated by either L-type Ca^2+^ channels or the Na^+^/Ca^2+^ exchange mechanism. Comparison of the families of currents in the left versus the right columns confirms that this maneuver did block virtually all of the quasi instantaneous current changes. The P2, voltage clamp steps (as described in detail in the text) were applied in an attempt to demonstrate the approximate values for the reversal potential of the I_f_ currents that were activated by the hyperpolarizing P1 voltage commands.

The two separate sets of experimental data shown in Figure 4 were recorded at 35 °C during superfusion with normal Tyrode’s solution (left Panel), and in this solution after both BaCl_2_ (0.1 mM) and NiCl_2_ (0.5 mM) had been added to completely block any time-independent or background currents due to either inwardly rectifying K^+^ channels, or the non-inactivating component of the L-type Ca^2+^ current, respectively. In Figure 4 the superimposed families of I_f_ current records in both the left and right panels reveal the activation and time- and voltage-dependent development of I_f_ under baseline conditions (left); and after the contributions from I_K1_ or I_CaL_ had been blocked. The I–V relationships shown below each family of raw current records is based on data from three different myocytes. It is apparent from the results in Figure 4A that these types of recordings of I_f_ do indeed, exhibit a quasi-instantaneous current change; and that this is followed by much slower time- and voltage-dependent activation of I_f_. In contrast, the results in Figure 4C obtained after combined application of BaCl_2_ and NiCl_2_ confirm that a significant fraction of the quasi-instantaneous current is likely to be carried by I_K1_, I_CaL_ or both. 

In Figure 4A,C there is also information that confirms the approximate reversal potential of I_f_ under these conditions. A paired-pulse protocol (P1–P2 voltage clamp commands) was applied. This insight can be gained from inspection of the superimposed current changes that take place during each 500 ms P1 depolarizing clamp step. The P2 or second voltage clamp commands displaced the membrane potentials within the range −35 to +45 mV in 10 mV increments. Under baseline conditions shown in Figure 4A this confirmed that the reversal potential is near −15 mV +/− 10 mV. As shown in Figure 4C after BaCl_2_ and NiCl_2_ the reversal potential shifts somewhat, as do the kinetics of the I_f_ current records. In general, these results confirm that the ion selectivity for I_f_ under the conditions of our experiments is comparable to but perhaps somewhat more positive than the E_rev_ values reported previously [15,39]. 

### 2.2. Biophysical Analyses

A number of previous studies of I_f_ in isolated SAN tissue preparations and in single myocytes, as well as data sets obtained from studies based on heterologous expression of HCN channel isoforms (principally HCN4), have provided essential background information for our project. However, none of these studies include sufficient data concerning the steady-state voltage dependence and kinetics of I_f_ in adult mouse SAN to yield the following information:

1. The best fit descriptors for the kinetics of activation and deactivation of I_f_ within a wide range of membrane potentials, when the intracellular milieu is not altered by dialysis from the conventional patch electrode used for these recording.
2. The steady-state, as opposed to isochronal, voltage dependence for I_f_ in single myocytes at 35 °C.
3. The size (current density) and time course of I_f_ changes during the diastolic depolarization or pacemaker potential (approximately −70 to −50 mV).
4. Semiquantitative comparisons of the current changes due to I_f_ vs. those resulting from, e.g., deactivation of I_Kr_ and/or activation of I_CaL_. These current changes also occur during the development of each pacemaker depolarization, both at baseline and in the presence of physiological levels of ß adrenergic stimulation (1–10 nM ISO).

**Figure 5:**
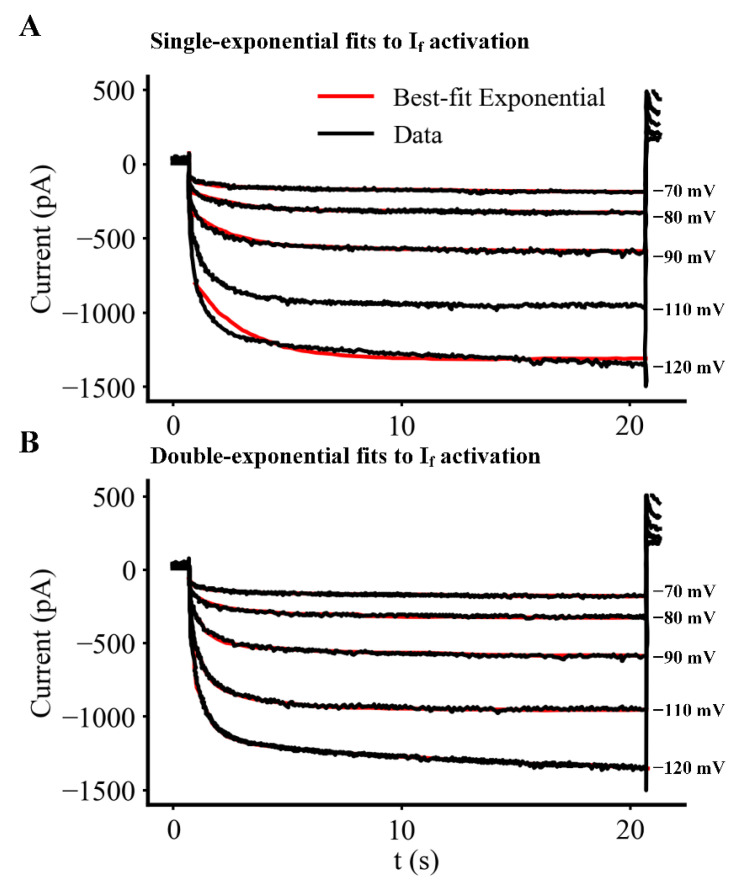
Analysis of the kinetics of activation and deactivation of I_f_ in an isolated adult mouse primary pacemaker myocyte. The kinetics of activation and deactivation of I_f_ as a function of membrane potential were determined using a family of 20 s rectangular voltage clamp steps. To study activation, hyperpolarizing steps from −70 to −120 mV from a holding potential of −35 mV were applied. For deactivation, a 10 s step to −120 mV was utilized to fully activate I_f_; and then its deactivation kinetics were studied by applying 10 s steps to membrane potentials in the range −50 to −110 mV. As shown, (red traces superimposed on black original current change records) when individual traces in these families of I_f_ currents were fitted using a function consisting of either one (**A**), or the sum of two (**B**), exponential relationships; the double exponential function provided a better fit (particularly to the onset kinetics). Similarly, the time courses of the deactivating records for I_f_ were better described by double exponential than by single exponential functions (data not shown). Additional details are provided in the Results and in the Supplemental data section.

**Figure 6:**
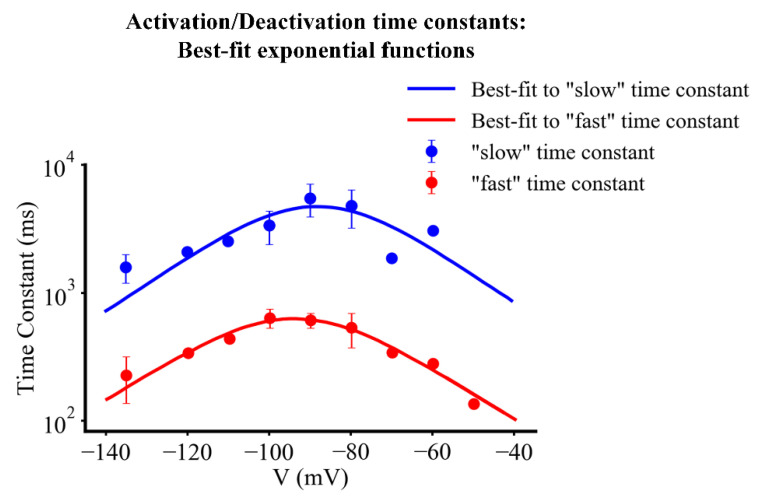
Plots of the voltage dependence for both the slow and the fast kinetic processes that underlie the activation and deactivation of I_f_. As indicated in the inset, data depicting the fast time constant for I_f_ kinetics is shown in red while data based on the determination of the slow time constant at each membrane potential is shown in black. Activation and deactivation time constants obtained at the same membrane potential have been pooled to form each of these plots. Details about the fit functions for the fast and slow time constants are given in Equations (S5) and (S6) in the Supplementary Materials.

Our analysis of the time courses for activation and deactivation of I_f_ as a function of membrane potential is presented in Figure 5 and Figure 6. Figure 5A,B consist of the same five I_f_ records, each elicited by long (20 s) hyperpolarizing voltage clamp steps from the holding potential (−35 mV) to the membrane potentials shown at the right of each current trace. To study the deactivation of I_f_, a 20 s voltage step to −120 mV from −35 mV was applied to fully activate I_f_. Deactivation kinetics of I_f_ could then be studied by immediately applying 10 s voltage steps to membrane potentials in the range −50 to −110 mV. In each panel of Figure 5, red curve-fitted relationships have been superimposed on the black original current change records. In Figure 5A, a single exponential function was used in an attempt to fit these I_f_ activation traces; in contrast, in Figure 5B the double exponential function provided below was used. It is apparent that the double exponential function provides a much better fit. 

This analysis of I_f_ kinetics yielded the aggregate data shown in Figure 6. Here, both the fast (red dots) and the slow (blue dots) time constants that combine to regulate the activation and deactivation of I_f_ are plotted as a function of membrane potential. For each kinetic process best fit functions were obtained. Details concerning the fitted functions for the fast and slow time constants and their relative ratio are given in Equations (S5), (S6) and (S9) in the Supplementary Materials.

This data set provides an initial insight that guided the emphasis of the remainder of this study. Note, from the data points corresponding to the faster of the two time constants that regulate I_f_, that in the pacemaker depolarization range of membrane potentials even this time constant ranges from approximately 100 to 300 ms. This information, applied in conjunction with the results in Figure 1 in which the cycle length of the spontaneous pacemaker has a mean value of 360 +/− 27 ms, reveals that during each interbeat interval the changes in I_f_ would be expected to be very small. Moreover, when the SAN beating rate increases significantly (30% to 70%) in the presence of ISO the time-dependent changes in I_f_ during the shortened diastolic intervals may be even smaller. That is, the activation of I_f_ that occurs during each action potential does *not* fully deactivate within the following diastolic interval. This ‘residual activation’ of I_f_ produces an almost time-invariant inward current lasting throughout the interbeat or diastolic interval, as well as large changes during the action potential.

**Figure 7:**
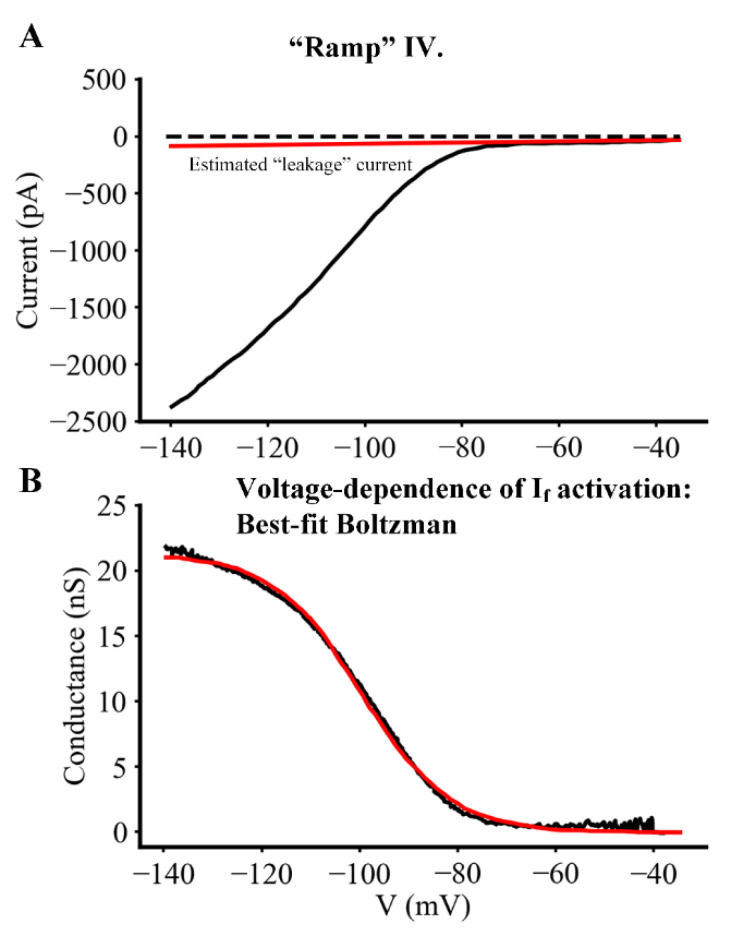
Determination of steady-state voltage dependence of activation of the transmembrane current, I_f._ Steady-state I–V relations for I_f_ were recorded, and the voltage dependence of activation for I_f_ was derived from primary data as described in Results. (**A**) Representative raw data of I–V relationship displayed as a signal averaged (*n* = 4). (**B**) Conductance values converted from I_f_ currents using the equation given in Results (assuming that the instantaneous I–V relationship for I_f_ is linear). The resulting conductance-voltage relation was fitted to a Boltzmann function (defined as: G = G_max_/(1 + exp[(V_m_ − V_h_)/s])) and plotted as shown in Panel B (red).

Further analysis of I_f_ biophysical properties focused on obtaining the relationship that governs the steady-state voltage dependence for the activation of this current. Applying voltage clamp protocols that utilized ramp, as opposed to rectangular, command waveforms made it possible to obtain this information much more quickly than when using a rectangular clamp step protocol. A ramp command waveform was combined with signal averaging to achieve the resolution needed to identify very small but functionally important changes in I_f_ that may contribute to each diastolic depolarization or pacemaker potential. The voltage ramp protocol was designed based on the kinetic data shown in Figure 6; it consisted of a linear ‘ramp’ that first depolarized the membrane potential from the holding potential of −60 mV to −35 mV; and then ‘ramped’ back to −135 mV over a period of 60 s. This was followed by an abrupt return to the holding potential of −60 mV. Application of this voltage clamp protocol resulted in a continuous current change such as the one shown in the black trace in Figure 7A. This ramp command was repeated four times at a rate of one per five minutes and the resulting current change records were averaged, yielding data sets such as the one shown in Figure 7A. 

To obtain information concerning the conductance-voltage relationship for I_f_, the small background current due to leakage through the amphotericin-mediated patch/myocyte surface membrane seal resistance was estimated and subtracted. A typical estimated leakage current, assuming a seal resistance in the 10 Giga-ohm range is shown in red in Figure 7A (assuming that this current is linear over the voltage range of interest). The red trace was subtracted from the black trace to yield the net I_f_ current voltage relationship. I_f_ values were then converted to conductance using the equation below assuming that the instantaneous I–V for I_f_ is linear:

G(V_m_) = I_f_/(V_m_ − V_rev_) (1)

where G is the conductance at each membrane potential, V_m_; and V_rev_ is the reversal potential of I_f_ (assumed to be −35 mV). As illustrated in Figure S7B and further specified in the Supplement Section, this conductance–voltage relationship can be fitted to a Boltzmann function of the form shown below:

G = G_max_/(1 + exp[(V_m_ − V_h_)/s]) (2)

where G_max_ denotes the ‘fully activated’ conductance, V_h_ is the membrane potential for half-activation of I_f_, and s is a slope factor at the membrane potential, V_h_. The best fit Boltzmann function (red trace in Panel B) had a G_max_ of 21.3 nS, V_h_ of −99.8 mV, and a slope factor, s, of 9.1 mV. We note, however, that a significant number of the steady-state current voltage relationships obtained for I_f_ failed to show the saturating characteristic depicted by the raw data in Figure 7B. When this was the case, it was not possible to use the best fit procedure to Equation (1). The reasons for this are unknown. However, a likely contributor is the fact that at extreme hyperpolarized potentials the exceptionally large currents due to strong, unphysiological activation of I_f_ result in intracellular increases in the concentration of one of the permeant ions, e.g. Na^+^. This possibility has been suggested in a number of previous publications and its consequences have been explored in detail in a recent paper addressing the hypothesis that strong activation of I_f_ may significantly alter [Na^+^]_i_ in isolated adult mouse SAN myocytes [40].

### 2.3. The Physiological Role of I_f_ in Mouse SAN Pacemaker Activity

**Figure 8:**
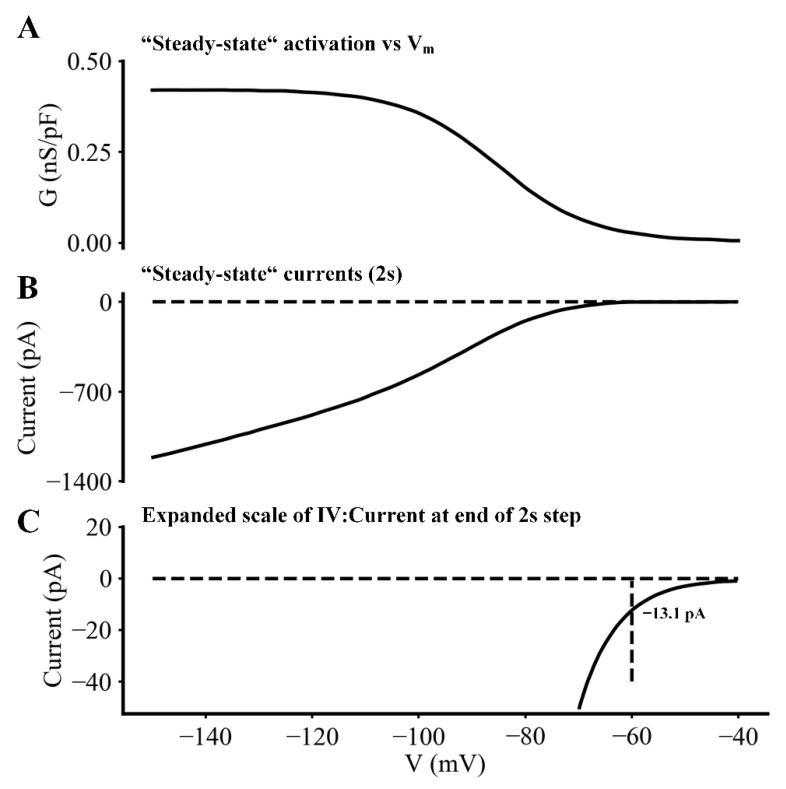
Current changes due to I_f_ during the pacemaker depolarization obtained using amphotericin-mediated patch electrode recordings at 35 °C in an adult mouse SAN pacemaker myocyte. (**A**) Steady-state activation relationship for I_f_ plotted as a function of membrane potential. (**B**) Illustration of the I_f_ current activated by the same voltage ramp protocol described in the Legend of Figure 7. (**C**) Plot of the same data as that in (**B**), after an approximately 30-fold increase in gain. This provides an indication of the maximal I_f_ current change in I_f_ that can be expected to occur within the range of membrane potentials (−40 mV to −70 mV). This range corresponds to the membrane potentials during the spontaneous pacemaker depolarization (see Figure 1A).

The final two sets of experiments were carried out in an attempt to obtain sets of current changes due to I_f_ at sufficiently high resolution that may yield insights into the functional roles of selected ion channel fluxes and net current changes during the pacemaker depolarization. In the experiments illustrated in Figure 8 (carried out under baseline conditions and also in the presence of ISO) a triangular shaped voltage clamp command waveform was applied from a holding potential of −40 mV. This waveform consisted of a linear hyperpolarizing ramp to −140 mV from −40 mV over a 2 s time period. This waveform was applied once every 20 s. A typical steady-state current trajectory in this range of potentials is shown in Figure 8B and the conductance voltage relationship derived from this data is shown in Figure 8A. Inspection of the information in Figure 8A,B appears to reveal a small conductance increase within the pacemaker range of potentials (−60 to −40 mV), but the underlying current change is not well resolved, even in the signal averaged traces shown in Figure 8B. However, when these same current changes are plotted at much higher resolution (as shown in Figure 8C), it is apparent that within the voltage range −40 to −60 mV an inward current measuring approximately −13 pA does develop in this ‘pacemaker range of potentials’. This is of interest and importance since the size of the net inward current that is needed to drive the pacemaker depolarization in a 30 pF myocyte is estimated to be only 1.0 to 1.5 pA per SAN myocyte. This small net inward current can be calculated from the product of the myocyte capacitance (approximately 30 pF) and the rate of change of the membrane potential (0.031 volts/s) during the pacemaker depolarization. However, we note that this small increase in inward current due to I_f_ always develops in the presence of: (i) a larger deactivation of outward current carried by I_Kr_ [8]; and also (ii) a significant time- and voltage-dependent increase in I_CaL_ that is evident in the last one third of the pacemaker depolarization.

**Figure 9:**
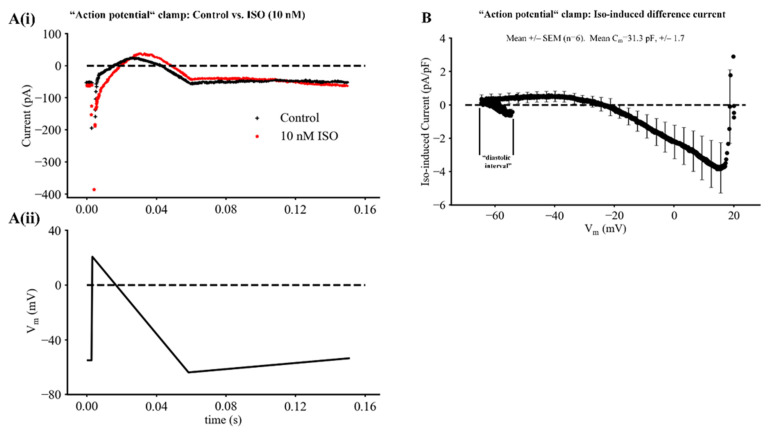
Ramp voltage clamp procedure to reveal the ISO-induced current changes during the action potential and pacemaker depolarization in adult mouse SAN. (**A**) Superimposed current records, each obtained after averaging 6 current traces generated by repetitive application of the action potential-like voltage ramp waveforms shown in Panel Aii. The baseline or control record is in black and the analogous averaged record obtained after 10 nM ISO application is in red. (**B**) Mean I-V relation for the ISO-induced current obtained as an averaged record from six different SAN myocyte recordings. Note: (i) the large increase in net inward current in the voltage range approximately +20 to −20 mV likely due to ISO-induced augmentation of I_Ca-L_;) and (ii) that ISO at 10 nM also resulted in a consistent but very small increase in net outward current at membrane potentials between −20 and approximately −65 mV (see Section 4).

An additional analysis of the transmembrane current changes responsible for the SAN pacemaker depolarization is illustrated by the averaged results shown in Figure 9. In these experiments, the ramp voltage clamp waveform shown in Panel Aii was applied repetitively from a holding potential of −60 mV. The SAN myocyte was initially rapidly depolarized to +20 mV before membrane potential was then returned to −60 mV driven by a linear ramp waveform lasting 600 ms, and then slowly depolarized over a 1 s time period from −60 to −40 mV. After baseline data (black) was collected, 10 nM ISO (red) was applied. A typical raw data set is shown in Figure 9A. 

During the 600 ms triangular voltage stimulus, a transient inward current developed, although this is not well resolved in these recordings. Following this, a time- and voltage-dependent outward current was activated. It peaked and then declined in response to the slow repolarizing voltage command. After the membrane voltage was returned to approximately the maximum diastolic potential; a slow ramp depolarization was applied to mimic some aspects of the pacemaker depolarization. This resulted in a small decrease in this outward current as we have described previously [8]. This figure also shows the most significant changes that developed when this same hybrid ramp voltage clamp waveform was applied approximately 2 min after 10 nM ISO had been applied to the superfusate (red trace). The major ISO-induced differences included: (i) the development of a relatively long lasting net inward current. This was likely due to the ISO-induced augmentation of I_CaL_, although a small contribution from the late or slowly inactivating component of Na^+^ current cannot be excluded [38]. In addition, (ii) the two superimposed current traces in Figure 9A also reveal an ISO-induced enhancement of outward current and the slow decline of this current during the applied ‘pacemaker depolarization’. This current change is likely to be due to an ISO-induced enhancement of I_Kr_, although contributions from I_Ks_ cannot be ruled out in this experiment protocol [8]. 

The final part of our experimental study attempted to bring out the changes in I_f_ that are produced by the relatively low concentrations of ISO (1–10 nM). The results in Figure 9B are based on the average of the original recordings from six different SAN pacemaker myocytes, each driven or stimulated by the ramp voltage clamp protocol shown in Figure 9A. ISO-induced difference currents were obtained by subtracting the averaged records recorded under baseline conditions from those after ISO application. Note, that the main effects of this relatively strong ß adrenergic stimulation were: (i) a relatively large and long-lasting increase in net inward current occurring between the membrane potentials of approximately +20 and −20 mV. This difference is likely to have been produced by the well-known effects of ISO on I_CaL_. (ii) In addition, between the membrane potentials of approximately −20 and −60 mV (the maximum diastolic potential), the ISO-induced difference current was net outward, but also was very small compared to the preceding ISO-induced inward current change. (iii) Finally, in response to the imposed slow depolarization from the ‘maximum diastolic potential’ (−60 mV) to the ‘threshold’ (approximately −40 mV) for initiation of a SAN action potential, this ISO-induced difference current switched from being net outward to net inward. However, this change proved challenging to resolve even with the assistance of signal averaging of the raw current records. As illustrated in Figure 9B, the maximum value of this net current change averaged only approximately 0.3 to 1.0 pA/pF. Nonetheless, as previously pointed out, this very small net current is sufficient to strongly modulate the rate of the spontaneous pacemaker depolarization in a single myocyte that has a capacitance of approximately 30 pF. In fact, a net inward current of this size would be expected to at least double the rate of firing of the SAN myocyte under these experimental conditions (see Section 4).

### 2.4. Computational Analyses Based upon Simulations of SAN Pacing and Action Potentials

**Figure 10:**
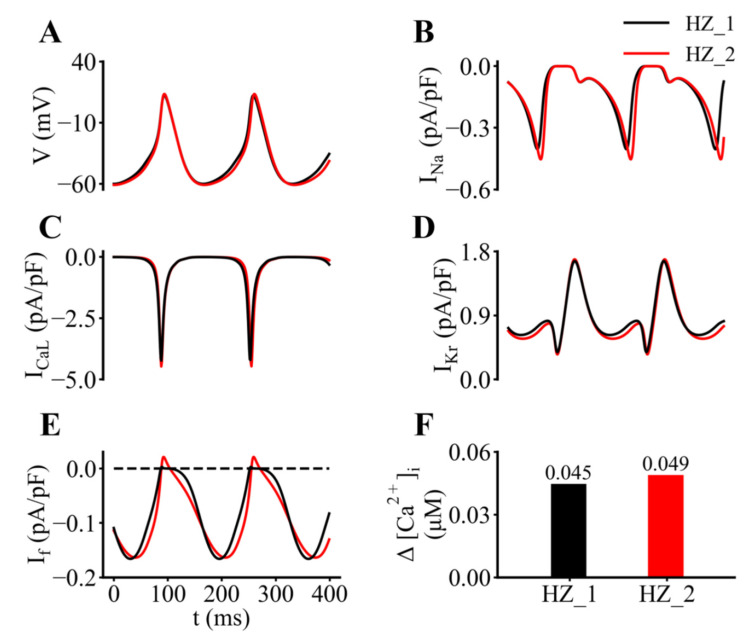
Mathematical simulations of SAN pacemaker activity which compare output from our original model (HZ-1) with that from the updated model (HZ-2) used in this study. In this figure, data from the original model [8,38] is shown in black (HZ-1) and output from the updated model (HZ-2) is shown in red. (**A**) Two superimposed traces of pacemaker depolarization and action potential cycles computed from HZ-1 and HZ-2 models. (**B**–**E**) Corresponding data for the Na^+^ current, I_Na_; the L-type Ca^2+^ current, I_CaL_; the rapid delayed rectifier K^+^, I_Kr_ current, and the hyperpolarization-activated cation current, I_f_, respectively. (**F**) Changes in intracellular Ca^2+^ measured as the difference between diastolic and systolic levels.

An important component of our analysis of the role(s) of I_f_ in generating the pacemaker potential in a myocyte from the adult mouse SAN was based on mathematical modeling of the pacemaker depolarization and action potential. As described in the Methods and documented in the Supplement section, this mathematical modeling was based on our original publication [38]) and also included our previously published modifications of the equations for the K^+^ current, I_Kr_ [8]). Representative output that characterizes the control or baseline data generated by the updated model, which exhibits an intrinsic firing frequency of approximately 6 Hz (HZ-2 model) used in this study is shown in Figure 10. In each panel of this figure, the superimposed red and black traces provide an indication of the small changes in each of these dynamic parameters that are introduced by updating the original Hu et al. [8] model (referred to as HZ-1 model) based on the new experimental data sets for I_f_ that we obtained (Figure 1, Figure 2, Figure 3, Figure 4, Figure 5, Figure 6, Figure 7 and Figure 8). In Figure 10 the individual panels show the basic electrophysiological activity (Figure 10A); and underlying changes in Na^+^ current (Figure 10B); L-type Ca^2+^ current (Figure 10C); rapid delayed rectifier K^+^ current, I_Kr_ (Figure 10D) and hyperpolarization-activated cation current I_f_ (Figure 10E). The histograms in Figure 10F of Figure 10 summarize the changes in [Ca^2+^]_i_, measured as the difference between the systolic and diastolic levels generated by the original (HZ-1) and the updated (HZ-2) models. In all cases these comparisons were based on computations carried out under steady-state conditions. Inspection of these results reveal that there are detectable changes in I_f_, as well as changes in I_CaL_ and I_Kr_ arising from secondary effects of altered pacemaking action potentials. In particular inspection of I_f_ records showed that changes in this current during the action potential occur simultaneously with a steady inward current of about 0.15 pA/pF. 

After gaining these insights into the behaviour of our modified model at baseline (or in control settings) the electrophysiological characteristics of a spontaneously active adult mouse SAN myocyte, in response to application of 10 nM ISO were explored. Our approach for obtaining this information involved exploring the dose-dependent modulations by ISO on the maximal conductance parameters and/or the position of the steady-state activation curves for I_f_, I_CaL_, I_CaT_, I_st_, I_Kr_ and I_Ks_. In addition, although Ca^2+^ homeostasis was not emphasized in this study, the model parameters that regulate intracellular Ca^2+^ buffering and release were monitored. Details of relevant equations and parameters for ISO-induced changes in these ion channels (and the intracellular Ca^2+^ handling) are provided in the Supplement Section.

**Figure 11:**
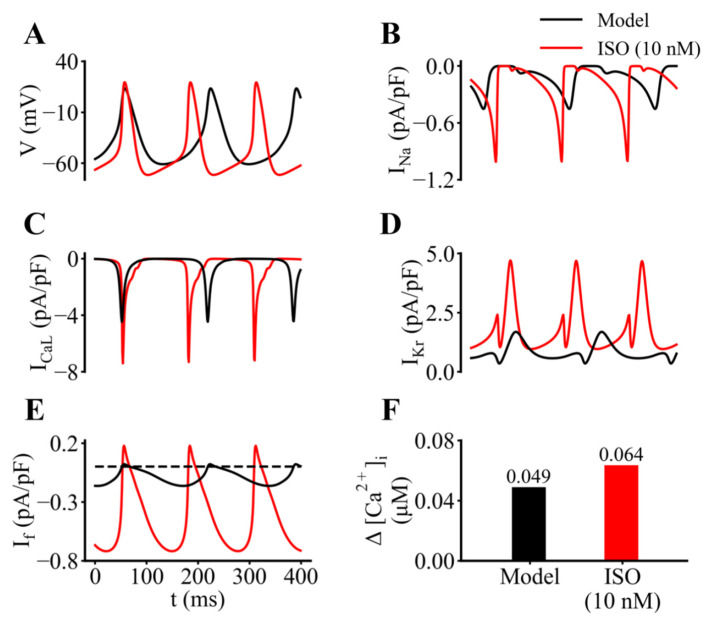
Illustration of the effects of ISO on SAN pacemaker activity and underlying transmembrane ionic currents. Output records generated using the baseline mathematical model is shown in black and data obtained after addition of 10 nM ISO is shown in red. (**A**) Superimposed action potentials and pacemaker depolarizations confirm that ISO application results in an approximately 50% increase in pacemaker firing rate, and also reveal a small hyperpolarization of the maximum diastolic potential. (**B**–**E**) Corresponding data for the Na^+^ current, I_Na_; the L-type Ca^2+^ current, I_CaL_; the rapid delayed rectifier K^+^, I_Kr_ current, and the hyperpolarization-activated cation current, I_f_, respectively. Note that application of ISO approximately doubles the size of I_Na_ and I_CaL_ and that I_Kr_ also increases significantly. The data in Panel C is the focus of this study. Two prominent effects of ISO on I_f_ are shown: (i) there is a significant shift in the inward direction of the steady or background current level, and (ii) the changes in I_f_ during each action potential are increased substantially (4- to 5-fold). (**F**) Increase (approximately 30%) in intracellular Ca^2+^ by ISO.

Some of the most interesting findings from this in silico analysis are summarized in Figure 11 in which control or baseline data sets (black) generated by our updated model are directly compared with analogous results obtained after the application of 10 nM ISO (red traces). As expected, the SAN myocyte firing rate in ISO is increased (approximately 30%; see Figure 11A). Comparisons of the underlying transmembrane currents also reveal significant increases in the Na^+^ current (Figure 11B), L-type Ca^2+^ current, and delayed rectifier K^+^ current, I_Kr_. Note also that the hyperpolarization-activated cation current, I_f_, is increased substantially both in terms of there being a larger maintained inward current and the changes in I_f_ generated by each action potential being much larger. Approximate indications of the diastolic and systolic changes in [Ca^2+^]_i_ are illustrated in Figure 11F. 

From this pattern of results, it is apparent that the two largest ISO-induced current changes during the diastolic depolarization are those due to I_CaL_ and I_Kr_. It is important to note, however, that in relative terms there are equally large changes in I_f_. Careful inspection of Panel E reveals that the background or steady component of I_f_ has increased approximately 3-fold and in addition, the transient component has increased at least 4-fold (see Section 4). 

Additional data that document and further illustrate these findings are presented in two of the tables in the Supplement section. Table SV summarizes our computational results obtained by simulating the effects of 10 nM ISO on SAN electrophysiological responses in both the original (HZ-1) and the updated (HZ-2) models. These compare favourably to our experimental results shown in Figure 1. Table SVI in the Supplement Section provides data concerning the dose dependence of the effects of ISO on these parameters or biomarkers.

## 3. Discussion

### 3.1. Experimental Data

Results from this study confirm that our experimental methods [8,23] for localization and dissection of the primary pacemaker region of the adult mouse SAN, and enzymatic myocyte dispersion procedures can consistently yield the small numbers of spontaneously active myocytes that are required for detailed electrophysiological/biophysical studies. In the absence of autonomic tone, these isolated single SAN myocytes in normal Tyrodes superfusate pace or fire at a rate between 2.5 and 3 Hz at 35 °C. In addition, a significant fraction (50% to 60%) of them have the membrane properties that are needed for application of amphotericin-mediated patch clamp methods. Importantly, most of the data sets in this paper and results from our previous experimental studies [23] and mathematical modeling [8] are quite consistent with results from previous comprehensive studies aimed at defining the key electrophysical properties of isolated myocytes from the anatomical adult mouse SAN [22,27]. These data sets and results in this paper also provide a basis for analysis of any apparent differences between the procedures that were used, or functional properties of the resultant myocyte populations. 

Our ability to record at 35 °C, combined with the minimal dilution of the myoplasm or dialysis with the intra-pipette solution, made possible by amphotericin-mediated patch methods also contributed to our preliminary analysis of some aspects of β adrenergic stimulation following bath application of 1–10 nM ISO. As expected, a 2–3 fold increase in L-type Ca^2+^ current in response to, e.g., 5 nM ISO was consistently observed. This classical response c.f. [20] provided a firm basis for a comparison of the β adrenergic effects of ISO on I_CaL_ vs. on I_f_ in our study.

Detailed analysis of the physiological, pharmacological and biophysical properties of I_f_ at 35 °C was the main focus of the experimental component of this study. The time- and voltage-dependent properties of I_f_ that are shown in Figure 5 and Figure 6, when considered in conjunction with the steady-state voltage dependence of this current/conductance (Figure 7) provide this information. These findings complement those in the recent publication by Peters et al. [39]. Results from that study emphasize that in order to fully understand the functional consequences of changes in I_f_ in SAN myocytes, it is essential to recognize that only 2% to 5% of the maximal I_f_ conductance needs to be activated. Based on their analysis, Peters et al. [38] have also pointed out that the quasi instantaneous component of If needs to be accounted for when analyzing the role of If in the pacemaker depolarization. Our findings (Figure 4, Figure 10 and Figure 11) support these conclusions. In addition, we reveal and illustrate the importance of these small changes in I_f_ in the pacemaker range of potentials both under baseline, or control, conditions and in the presence of ISO (Figures S6 and S9). 

In fact, most previous recordings of I_f_, whether based on data from isolated SAN tissue experiments or single myocyte recordings, have clearly revealed a significant, very rapidly activating or quasi instantaneous component of I_f_ c.f. [3,4,15,16,17]. This component of I_f_ has also been identified and studied in detail in heterologous expression/patch clamp studies of both of the principal transcripts (HNC4 and HCN2) that generate I_f_ in mammalian hearts [41,42]. We note, however, (and illustrate in Figure 4) that in many cases, conventional rectangular voltage clamp hyperpolarizations of single myocytes can give rise to very rapidly activating inward currents that are not generated entirely by I_f_. Instead, this quasi-instantaneous current change includes significant contributions from the inwardly rectifying background K^+^ current, I_K1_, as judged from the reduction of this component by BaCl_2._ Slowly inactivating or non-inactivating inward currents contributed by either L-type Ca^2+^ channels or Na^+^ currents may also contribute to the quasi-instantaneous current changes [13,43,44], particularly when isolated SAN myocytes exhibit the ‘follower phenotype’, i.e., are derived from peripheral locations within the anatomical mammalian SAN.

A more complete understanding of the basis for this quasi-instantaneous hyperpolarization-activation inward current will require additional experiments, including careful delineation of the central vs. the peripheral, or primary vs. follower pacemaker region, of the anatomical SAN. This type of analysis and important differences in spontaneous pacemaker activity and underlying transmembrane currents have been reported for myocytes isolated from the entire or anatomical, rabbit SAN [43,44,45]. Functionally, important aspects of these differences have been replicated using mathematical modeling [46]. Somewhat similar data describing progressively larger expression levels for the nonlinear background K^+^ current, I_K1_, in myocytes from the adult rat SAN [47] and within human SAN tissue have also been reported [7,48].

### 3.2. Mathematical Modeling

We have attempted to integrate our experimental findings and analyze their main implications by modifying and making use of a mathematical model of the electrophysiological activity of spontaneously active adult mouse SAN myocytes. This ‘parent model’ was originally developed by Kharche et al. [38]. It was updated previously based on our analysis of the biophysical and pharmacological properties of the rapidly activating delayed rectifier K^+^ current, I_Kr_, [8]. Before the mathematical simulations that are the basis for Figure 10 and Figure 11 were carried out, the equations for I_f_ were modified based on our experimental findings shown in Figure 1, Figure 2, Figure 3, Figure 4, Figure 5, Figure 6, Figure 7, Figure 8 and Figure 9. Additional changes to the original model [38] were also made, guided by the results in this paper (e.g., Figure 2, Figure 3, Figure 9, Figure 10 and Figure 11) as well as Tables SIII and SIV in the Supplemental section) obtained in the presence of ß adrenergic stimulation resulting from low doses (1–10 nM) of ISO being added to the superfusate. As shown in Figures S5–S9 in the Supplement Section, our model can provide a useful platform for comparing and contrasting some aspects of the functional roles of changes in individual transmembrane ionic currents that underlie both the pacemaker depolarization and the membrane action potential components of myogenic pacing in the adult mouse SAN myocyte. Somewhat similar mathematical modeling of the positive chronotropic effects of β adrenergic stimulation in the adult rabbit SAN [29,49] as well as in human SAN c.f. [20,50] have previously been published. 

Mathematical modeling can provide a basis for detailed inspection of the net ionic current changes that underlie development of the pacemaker potential. In fact, in the mammalian SAN mathematical simulations are essential for this type of analysis since the net current change generating a pacemaker depolarization (having a dV/dt of only approximately 0.1 V/s) is expected to be only in the 0.5 to 2.0 pA per myocyte range. A requirement for this high resolution presents a significant experimental challenge, even when methods such as signal averaging are employed to improve the signal-to-noise ratio c.f. [51]. As mentioned, the recent paper by Peters et al. [39] provides complementary data and offers an independent analysis of I_f_ in isolated myocytes from mouse SAN in the presence of 5 nM ISO.

In mammalian SAN at least three different currents (I_CaL_, I_Kr_, and I_f_) change substantially and interact to result in the small net inward current that drives the slow diastolic depolarization. The experimental data sets in Figure 8 and Figure 9 illustrate our efforts to identify the net current changes that underlie the action potential and the pacemaker depolarization in the adult mouse SAN, both at baseline and in the presence of ISO. Additional data sets are needed to provide a basis for convincingly separating these small net current changes into individual components generated by, e.g., I_f_, I_CaL_, and/or I_Kr_. We acknowledge also, that in the mouse SAN changes in both T-type Ca^2+^ current [52] and the cardiac isoform of the Na^+^ current [53,54] can alter heart rate and may be involved in generation of the pacemaker potential. The preliminary results based on mathematical modeling shown in Figure 10 and Figure 11 represent a useful starting point in this analysis. Further insights into this multifactorial problem will require new experimental data, as well as implementation of ‘population of models’ approaches that we and others have used to advantage [55,56].

### 3.3. Physiological and Biophysical Insights

The most significant mechanistic insight from this study is that there often is an apparent discordance, or mismatch, between the macroscopic time- and voltage-dependent kinetics that regulate I_f_, and the presence of a very small but functionally important component of this current that is present (see Figure 9, Figure 10 and Figure 11) during the diastolic interval of adult mouse SAN pacemaker myocytes. Important aspects of this discrepancy have been identified and studied in the recent paper by Peters et al. [39]. As illustrated in Figure 5, our detailed study of the activation kinetics, complemented with an analysis of the deactivation kinetics of I_f_ showed that these kinetic processes are well described by functions consisting of the sum of two exponentials. However, this analysis (Figure 6) also revealed that neither of these time courses are fast enough to be able to fully account for the small but significant changes in I_f_ that take place within the very short diastolic intervals (150–200 msec.) that are characteristic of those in the adult mouse heart, either under control conditions or in the setting of adrenergic tone c.f. [20]. This insight and mathematical simulations of changes in I_f_ based on our experimental data (Figure 10 and Figure 11) when incorporated into our revised biophysical model of this current at 35 °C suggest that the quasi instantaneous component of I_f_ is of primary importance as a component of the net inward current that is essential for driving SAN pacemaker activity. Some aspects of this functional role have been recognized previously [57,58,59]; in fact, the quasi steady-state component of I_f_ has been denoted a ‘depolarization reserve’ in primary and secondary SAN pacemakers, as well as in the AV node of the mammalian heart. 

Inspection of Figure 10 and Figure 11C clearly shows that this ‘preactivated’ current due to I_f_ channel activity, driven by the changes in membrane voltage during the pacemaker depolarization and the action potential of the SAN myocyte, produces the rapid ‘ohmic’ current changes. Note that at a distinguishing feature of the preactivated component of I_f_ is that these changes very closely ‘mirror’ the time course of the membrane action potential, both at baseline and in the presence of ISO. Accordingly, it will be important to fully understand the functional role of this specific contribution of I_f_ to SAN pacemaker activity. Important new information that will be able to be generated in additional experimental and theoretical studies includes:

- Determining the exact position of the *steady-state* activation curve in relation to the maximum diastolic potential (MDP) of the SAN action potential in various translational contexts. This is because even very small alterations in the MDP, and/or +/−5 mV shifts in the midpoint of the *steady-state* activation relationship for I_f_ are likely to result in functionally important changes in the extent to which I_f_ is preactivated. Situations in which this residual activation of I_f_ is known to occur include: (a) changes in autonomic tone and related alterations in protein kinase A-mediated phosphorylation of I_f_ channels [20,60,61]; (b) alterations in intracellular cyclic AMP levels and attendant changes in I_f_ open probability [62,63,64]; (c) cyclical changes in I_f_ due to it being a target for circadian clock signaling [65,66]; (d) adaptations associated with healthy aging of the heart [67,68] or pregnancy [69].
- Revisiting and gaining an improved understanding of the ways in which changes in either extracellular or intracellular Ca^2+^ can act as a physiological stimulant. These changes can alter both components of I_f_ [70], or contribute to abnormal pacemaker activity by altering either transmembrane Ca^2+^ fluxes screening surface charges near I_f_ channels and/or altering intracellular signaling domains [71] or disturbing intracellular Ca^2+^ release or buffering [72].

Site-specific mutations in well-defined regions of the integral membrane protein complex that is responsible for generating I_f_ have been studied in detail and catalogued comprehensively [73,74,75,76]. Since a number of these ‘channelopathies’ exhibit a phenotype that is characterized by very large shifts in the voltage dependence for I_f_ activation, their functional consequences may need to be reconsidered in terms of specific effects on either the steady component of I_f_ or the component regulated by activation or deactivation kinetics. Similarly, when molecular pharmacological approaches are being considered or developed that target I_f_ it may be possible to selectively modulate the component due to the residual activation of this channel [77]. 

### 3.4. Closing Perspectives

When considering the translational significance of this work, it is important to place our results in a species-dependent context by recognizing that the exceptionally high heart rate of the adult mouse (550–600 bpm) has the consequence that in this species I_f_ may be expected to exhibit larger and rapidly changing current components than are present in the rabbit SAN (300 bpm) or in human SAN (60–80 bpm). This has been recognized in a recent paper from the Boyett group [77]. Contributions of the ‘preactivated’ or residual component of I_f_ to adult mouse SAN pacemaker activity will also need to be carefully considered in relation to the stimulus or pacing paradigm imposed on the experimental tissue or isolated myocyte preparations that are used for physiological, pharmacological or biophysical analyses [78]. This may include any experimental or clinical setting in which the heart rate is slowed dramatically, or perhaps even arrested, for periods of time lasting 10 to 30 min, (e.g., open heart surgery or cold storage of pacemaker tissue). Some of these situations have previously been considered in detail. For example, activity-dependent changes in intracellular Na^+^ [79] and subsequent, linked alterations in Na^+^/Ca^2+^ exchange or G-protein-mediated K^+^ channel function have been reported [80,81]. In addition, an interesting recent paper has drawn attention to the possibility that Na^+^ fluxes through activated I_f_ channels could also contribute significantly to intracellular Na^+^ homeostasis [40]. This possibility further detailed consideration of whether the quasi steady-state or the time- and voltage-dependent component of the Na^+^ fluxes through I_f_ can significantly change intracellular Na^+^ levels.

Our results also should be considered in relation to important features of the classical concept of ‘induced pacemaker activity’ [1,82]. In this paradigm, pacemaker activity is evoked by application of long lasting small depolarizing stimuli that are somewhat similar to the non-inactivating component of I_f_. Considerations such as this may provide a translational context as basic scientists and clinical investigators continue to seek improved mechanistic understanding of ‘cardiac automaticity’ [83], or consider the use of drugs that block I_f_ (such as ivabradine) to manage the global ischaemia associated with coronary artery disease or heart failure [84].

## 4. Material and Methods

### 4.1. Mouse SAN Myocyte Isolation and Patch Clamp Recordings 

The methods that we have used to: (i) dissect the sinoatrial node (SAN) tissue, (ii) locate the leading or primary pacemaker site, (iii) enzymatically isolate spontaneously active myocytes and place aliquots of them in a superfusion chamber, and (iv) make amphotericin-mediated patch clamp recordings at 35 °C +/− 1 °C have been described in detail in our previous publications [8,23]. All of our recordings were made four to seven hours after the enzymatic isolation of single myocytes from the SAN tissue was successfully completed.

### 4.2. Simulation Procedures

#### 4.2.1. The Parent SAN Myocyte Model

The mathematical model originally developed by Kharche et al. [38] and recently modified for our studies of I_Kr_, and related changes in the pacemaker potential and action potential in adult mouse SAN myocytes [8], was used as the starting point for this study. As indicated, in the present study, our main goal was to investigate in detail the functional role(s) of the hyperpolarization-activated cation current, I_f_, during spontaneous pacemaker depolarization in the adult mouse SAN. The Kharche et al. model [38] was chosen as a starting point based on its quite extensive validation against key features of experimental data describing most of the major transmembrane currents in mouse SAN myocytes. In the present study, the modifications to the equations for I_Kr_ that we have recently published [8] were utilized, as we attempted to accurately simulate each of the major transmembrane current changes that takes place in the range of membrane potentials that are spanned by the development of the pacemaker potential or slow diastolic depolarization. As described below, the extent to which these simulations can reveal important functional properties underlying mouse SAN pacemaking was assessed. Many of the key findings are presented in Figures S5–S9.

#### 4.2.2. Revision and Update of the Mathematical Descriptors for I_f_ in Mouse SAN Model

Our original model for the mouse SAN myocyte [38] included an informative, but relatively simple mathematical description of the kinetics and ion transfer relationship for I_f_. The experimental data in the present paper provides much more detailed information concerning the voltage dependence and kinetics of I_f_ at 35 °C. As reported recently [39] in mouse SAN the kinetics of I_f_ are somewhat complex. Both its onset and decay time courses are best described by an expression consisting of the sum of two exponential processes (see Results and also [39]). For these reasons the mathematical model developed for use in the present study includes a new set of equations and parameters derived by fitting the model equations to our experimental data for I_f_. This fitting procedure and optimization method has been published by Luersen et al. [85]. Detailed information concerning the new equations for I_f_ and relevant parameters are provided in Table SI, Figures S1 and S2. When simulating the actions of ISO, the same approach that we used in our previous study [38] was employed to account for the main effects of ISO. In brief, the maximal channel conductances of I_f_, the Ca^2+^ channel currents (I_CaL_, I_CaT_) and the sustained inward current (I_st_); as well as the maximal channel conductances of the K^+^ currents (I_Kr_, I_Ks_) were scaled as specified in Table SIII and Mathematical equations in Table SIV in the Supplement Section SII. The kinetics and rectifier ratio of I_Kr_ were also modified as illustrated in the Supplement Section SII. Small changes in the parameters that regulate intracellular Ca^2+^ handling were also made. Details of the ISO actions on the membrane ion channel currents and on the Ca^2+^ handling can be found in our previous paper [38] and in the Supplement Section SII. 

#### 4.2.3. Numerical Procedures and Schemes

The sets of equations that are the basis for the updated model were solved using a fixed time step of 1 × 10^−3^ ms. This was sufficiently small to ensure stable numerical solutions. Each simulation epoch consisted of computing a 20 s train of action potentials and pacemaker depolarizations together with continuous time records of the selected underlying ion channel-mediated currents. The last 1 s section of the data generated by each computational epoch was selected for presentation and analysis in each figure in this paper. 

## Data Availability

All data presented in this study are included in the main text and the Supplementary Materials.

## Supplementary Materials

The following supporting information can be downloaded at: https://www.mdpi.com/article/10.3390/ijms23084299/s1.
