# Supplementary material for: Frequency-Dependent Properties of the Hyperpolarization-Activated Cation Current, If, in Adult Mouse Heart Primary Pacemaker Myocytes
Source: Int J Mol Sci. 2022 Apr 13;23(8):4299. doi: 10.3390/ijms23084299 (PMC9024942; doi:10.3390/ijms23084299)
Supplement: Supplementary file 1 [file ijms-23-04299-s001.zip › ijms-1638941-supplementary.pdf]

Table of Contents for Supplement Section

SI. Model Development and Validation

- Table SI: Updated model equations for I_f (page S3)
- Figure S1: Experimental data for I_f superimposed on model output (page S4)
- Figure S2: Simulated I-V relationship for I_f (page S5)

SII. Mathematical Model Results for ISO-induced Changes in Mouse SAN Electrophysiological Activity and Underlying Currents

- Table SII: Summary of the effects of graded changes in the conductance of I_f in mouse SAN model using the revised model (page S6)
- Figure S3: Illustration of effects of graded block of I_f (page S7)
- Table SIII: List of parameter changes used to model ISO effects (page S8)
- Table SIV: Summary of equations used for ISO-dependent effects on SAN (pages S9 to S11)
- Table SV: Summary of the dose-dependent effects of ISO on SAN pacemaker parameters (page S11)
- Table SVI: Effects of 0.1 to 10 nM ISO on SAN pacing and model parameters (page Sl2)
- Figure S4: Histograms of 10 nM ISO effects on SAN pacing (page S13)
- Figure S5: Simulated results of the effects of 0.5 nM ISO on the main current
- changes that underlie the pacemaker depolarization (page S14)
- Figure S6: Simulated results of the effects of 0.5 nM ISO on the main current changes that underlie the pacemaker depolarization (page S15)
- Figure S7: Histograms depicting dose-dependent ISO effects on the pacemaker activity biomarkers (page S16)
- Figure S8: Illustration of the changes in pacemaker cycle length and underlying ionic currents produced by either 0.5 or 10 nM ISO (page S17)

SIII. Selected Changes in Intracellular Ion Homeostasis Induced by ISO in Mouse SAN

- Figure S9: Summary of changes in intracellular Na^{+} and Ca^{2+} homeostasis in response to 10 nM ISO (page S18)

Supplement Section

SI. Model Development and Validation

Table SI: Updated model equation for I_f

$$I_f = \sum_{i=1,2,4} \left(I_{fK_{hcni}} + I_{fNa\_hcni}\right) \quad (1)$$

$$I_{fK\_hcni} = g_{fk\_hcni}\left((1-f_V)\times p_{i,f} + f_V \times p_{i,s}\right)\times(V-E_K) \quad (2)$$

$$I_{fNa\_hcni} = g_{fNa\_hcni}\left((1-f_V)\times p_{i,f} + f_V \times p_{i,s}\right)\times(V-E_{Na}) \quad (3)$$

$$p_{i,f\infty} = p_{i,s\infty} = \frac{1}{1+e^{(V+100.27-S_{if\_iso})/9.73}} \quad (4)$$

$$\tau_{pi,f} = \frac{14.7}{e^{(V-0.77)/21.12} + e^{-(V+190.15)/21.88}} \quad (5)$$

$$\tau_{pi,s} = \frac{66.43}{e^{(V-7.59)/18.09} + e^{-(V+198.66)/22.71}} \quad (6)$$

$$\frac{dp_{i,f}}{dt} = \frac{p_{i,f\infty} - p_{i,f}}{\tau_{p_{i,f}}} \quad (7)$$

$$\frac{dp_{i,s}}{dt} = \frac{p_{i,s\infty} - p_{i,s}}{\tau_{p_{i,s}}} \quad (8)$$

$$f_v = \frac{\varphi_v}{1+\varphi_v} \quad (9)$$

$$\varphi_v = 1 \quad (10)$$

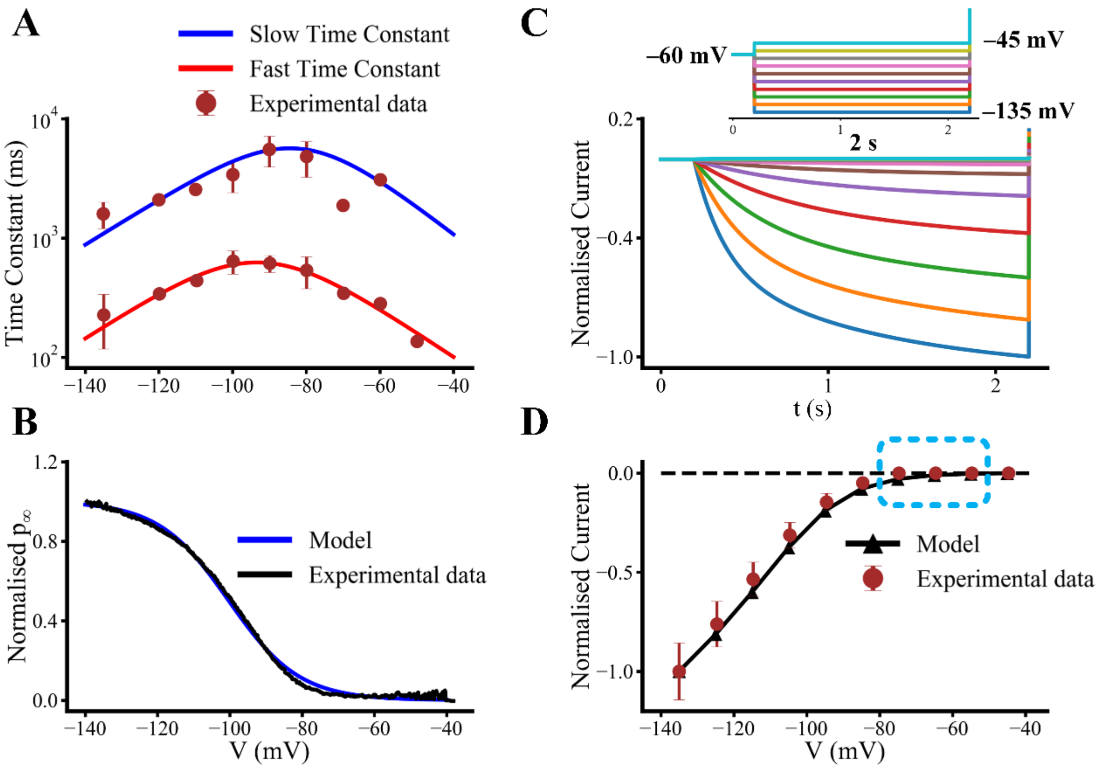

Figure S1. Simulated and experimental data of channel kinetics and I-V relationship. (A) Voltage-dependence of slow and fast time constants of I_f activation of I_f. (B) The steady-state activation curve of model (blue) and experimental data (black). (C) Time course of If traces during voltage-clamp protocol as shown in inset. (D) Comparison of simulated I-V relationship (black) with experimental data.

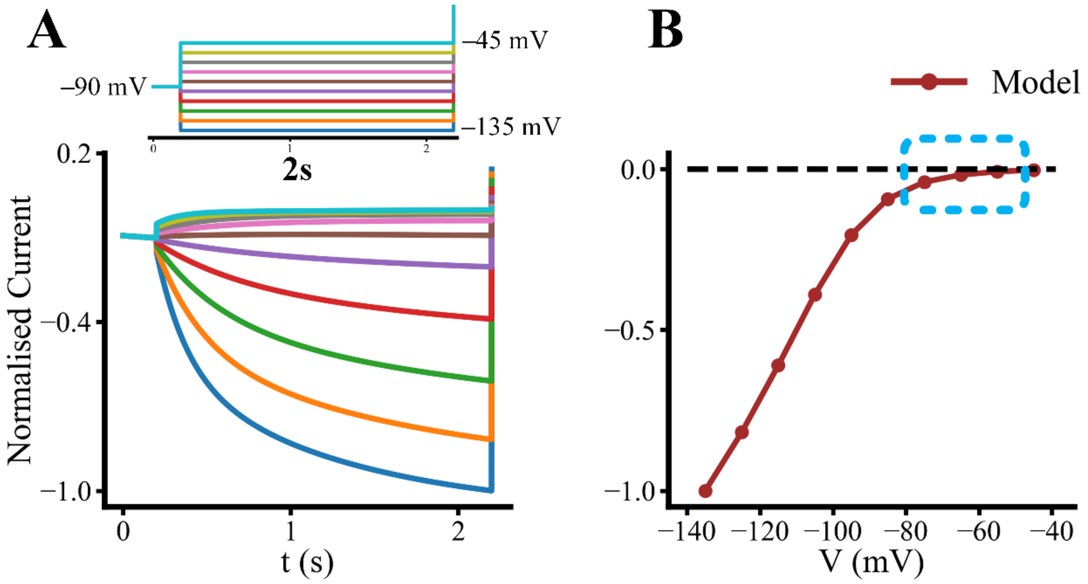

Figure S2. Simulated I_f records in response to the voltage-clamp protocol shown at the top of Panel (A). Panel B consists of isochronal (2 sec) I-V relationship. The range of membrane potentials that correlate with the pacemaker or diastolic depolarization is highlighted by the green rectangle.

SII. Mathematical Model Results for ISO-induced Changes in Mouse SAN Electrophysiological Activity and Underlying Currents

Table SII. Summary of the effects of graded changes in the conductance of I_f in the updated mouse SAN model.

Model		MDP (mV)	OS (mV)	dV/dt_{max} (V/s)	APD_{50} (ms)	APD_{90} (ms)	Pacing Frequency (HZ)	DDR (V/s)	TOP (mV)
CTR		–60.89	13.41	4.78	30.02	53.29	6.00	0.32	–39.30
g_f ×	0	-61.95	14.61	5.22	29.77	53.12	5.89	0.27	-44.19
	0.2	-61.72	14.35	5.13	29.82	53.16	5.92	0.28	-43.62
	0.4	-61.51	14.11	5.04	29.87	53.19	5.94	0.28	-42.95
	0.6	-61.30	13.87	4.95	29.92	53.23	5.96	0.29	-42.07
	0.8	-61.09	13.64	4.87	29.97	53.26	5.98	0.31	-40.73

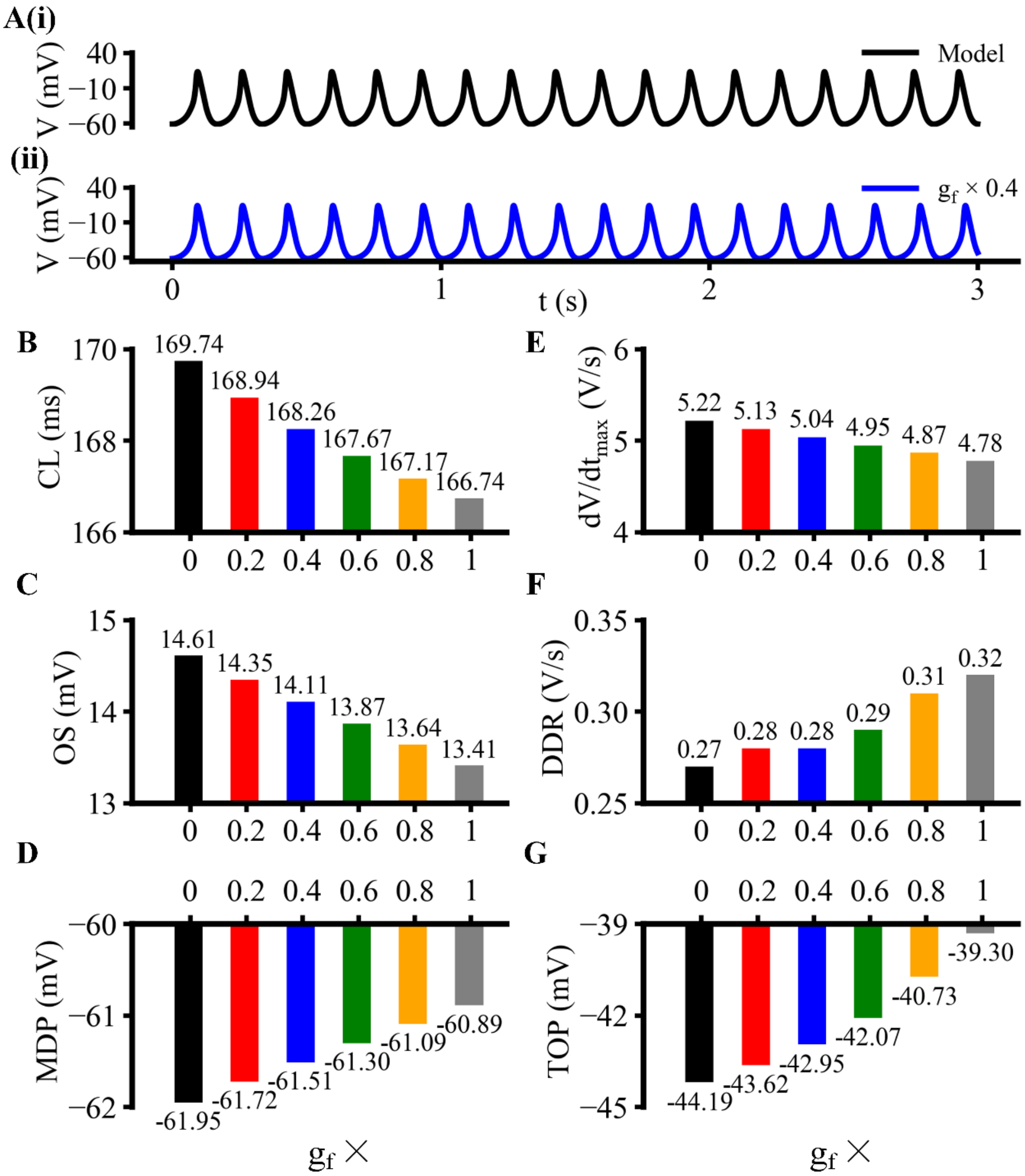

Figure S3. Illustration of effects of graded block of I_f (scale from 0 (control) to 1 (complete block) on the characteristics of pacemaking AP waveforms. (A(i)-A(ii)) Illustrative trains of APs for control (black) and g_{Kr} x0.4 (blue). (B) Cycle length (CL). (C) Overshoot potential (OS). (D) Maximal diastolic potential (MDP). (E) Maximal upstroke velocity (dV/dt_{max}). (F) Diastolic depolarization rate (DDR). (G) Take-off potential (TOP).

Table SIII. List of parameter changes used to model ISO (10 nM) effects

Ion channels		Maximal values of changes
I_{CaL}		maximum channel conductance increased by 1.9-fold
		steady state of activation and inactivation curves shifted by +10mV
I_{CaT}		maximum channel conductance increased by 2-fold
I_f		steady state of fast and slow activation curves shifted by –13.6mV
I_{Kr}		maximum conductance increased by 2.87-fold.
		steady state of fast and slow activation curves shifted by +15mV
I_{Ks}		maximum conductance increased by 2.87-fold
I_{st}		maximum conductance increased by 2-fold
Ca^{2+} handling in the SR.	k_{oSRCa}	k_{oSRCa} is doubled (k_{oSRCa}: Ca^{2+}-dependent RyR rate constant not limited by diffusion)
	K_{mf}	K_{mf} is reduced by 50% (K_{mf} : Forward-mode Ca^{2+} affinity of the SERCA pump)

k_{oSRCa}: Ca^{2+}-dependent RyR rate constant not limited by diffusion, which can influence the parameters: "Resting (R)" (Fraction of reactivated (closed) RyR channels) and "Resting inactivated (RI)"(Fraction of RyR inactivated channels).
K_{mf}: Forward-mode [Ca^{2+}] affinity of the SERCA pump, which can influence the parameter j_{up} (Ca^{2+} uptake flux from the myoplasm to the NSR).

Table SIV: Summary of equations used for ISO-dependent effects on SAN

$$iso = [Iso]\ in\ nM \tag{11}$$

(i) I_{CaL}

$$f_{CaL\_iso} = 0.9 \times \frac{iso}{1.5 \times 10^{-2} + iso} \tag{12}$$

$$I_{CaL,1.2} = (1 + f_{CaL\_iso}) g_{CaL,1.2} d_{L,1.2} f_{L,1.2} f_{Ca} (V - E_{CaL\_SAN}) \tag{13}$$

$$I_{CaL,1.3} = (1 + f_{CaL\_iso}) g_{CaL,1.3} d_{L,1.3} f_{L,1.3} f_{Ca} (V - E_{CaL\_SAN}) \tag{14}$$

$$S_{CaL\_iso} = 10.0 \times \frac{iso}{1.5 \times 10^{-2} + iso} \tag{15}$$

$$d_{L,1.2,\infty} = \frac{1}{1 + e^{-(V+3.0+S_{CaL\_iso})/5}} \tag{16}$$

$$f_{L,1.2,\infty} = \frac{1}{1 + e^{(V+36.0+S_{CaL_{iso}})/4.6}} \tag{17}$$

$$d_{L,1.3,\infty} = \frac{1}{1 + e^{-(V+13.5+S_{CaL\_iso})/6}} \tag{18}$$

$$f_{L,1.3,\infty} = \frac{1}{1 + e^{(V+35.0+S_{CaL\_iso})/7.3}} \tag{19}$$

(ii) I_{CaT}

$$f_{CaT\_iso} = 1.0 \times \frac{iso}{1.5 \times 10^{-3} + iso} \tag{20}$$

$$I_{CaT} = (1 + f_{CaT\_iso}) g_{CaT} d_T f_T (V - E_{CaT}) \tag{21}$$

(iii) I_f (22)

$$S_{if\_iso} = 13.6 \times \frac{iso^{0.392}}{(1.35 \times 10^{-2})^{0.392} + iso^{0.392}} \tag{23}$$

$$p_{if,i,f\infty} = p_{if,i,s\infty} = \frac{1}{1 + e^{(V+100.27-S_{if\_iso})/9.73}}\ (i = 1,2,4) \tag{24}$$

$(iv)\ I_{Kr}$

$$f_{K\_iso} = 1.87 \times \frac{iso}{1.9 \times 10^{-3} + iso} \tag{25}$$

$$I_{Kr} = (1 + f_{K\_iso}) g_{Kr} p_a p_i (V - E_K) \tag{26}$$

$$S_{Kr\_iso} = 15.0 \times \frac{iso}{7.5 \times 10^{-3} + iso} \tag{27}$$

$$p_{Kr,a,f\infty} = p_{Kr,a,s\infty} = \frac{1}{1 + e^{-(V + 21.17 + S_{Kr_{iso}})/9.76}} \tag{28}$$

$(v)\ I_{Ks}$

$$I_{Ks} = (1 + f_{K\_iso}) g_{Ks} p_a p_i (V - E_{Ks}) \tag{29}$$

(vi) I_{st}

$$f_{st\_iso} = 1.0 \times \frac{iso}{3.3 \times 10^{-2} + iso} \tag{30}$$

$$I_{st} = (1 + f_{st\_iso}) g_{st} d_{st} f_{st} (V - E_{ist\_SAN}) \tag{31}$$

$(vii)\ Intracellular\ Ca^{2+}\ handling$

$$f_{RyR\_iso} = \frac{1}{1 + e^{-2.55 \times (\log(iso) + 2.5)}} \tag{32}$$

$$k_{oSRCa} = k_{oCa\_SAN} \times (1 + f_{RyR\_iso}) / k_{CaSR} \tag{33}$$

$$\frac{dO}{dt} = (k_{oSRCa} \times [Ca^{2+}]_{sub}^2 \times R - k_{om} \times O) - (k_{iSRCa} \times [Ca^{2+}]_{sub} \times O - k_{im} \times I) \tag{34}$$

$$\frac{dI}{dt} = (k_{iSRCa} \times [Ca^{2+}]_{sub} \times O - k_{im} \times I) - (k_{om} \times I - k_{oSRCa} \times [Ca^{2+}]_{sub}^2 \times RI) \tag{35}$$

$$\frac{dR}{dt} = (k_{im} \times RI - k_{iSRCa} \times [Ca^{2+}]_{sub} \times R) - (k_{oSRCa} \times [Ca^{2+}]_{sub}^2 \times RI - k_{om} \times O) \tag{36}$$

$$\frac{dRI}{dt} = (k_{om} \times I - k_{oSRCa} \times [Ca^{2+}]_{sub}^{2} \times RI) - (k_{im} \times RI - k_{iSRCa} \times [Ca^{2+}]_{sub} \times R) \quad (37)$$

$$f_{PLB\_iso} = \frac{1}{1 + e^{-3.0\times(\log(iso)+2.2)}} \quad (38)$$

$$K_{mf} = K_{mf\_SAN} \times (1.0 - 0.5 \times f_{PLB\_iso}) \quad (39)$$

$$j_{up} = P_{up} \times \frac{({[Ca^{2+}]_i}/{K_{mf}})^{n_{up}} - ({[Ca^{2+}]_{up}}/{K_{mr}})^{n_{up}}}{1 + ({[Ca^{2+}]_i}/{K_{mf}})^{n_{up}} - ({[Ca^{2+}]_{up}}/{K_{mr}})^{n_{up}}} \quad (40)$$

Table SV. Summary of the effects of ISO (10 nM) on SAN pacemaker parameters

Model		MDP (mV)	OS (mV)	dV/dt_{max} (V/s)	APD_{50} (ms)	APD_{90} (ms)	CL (ms)	DDR (V/s)	TOP (mV)
Original (HZ_1)	CTR	–60.08	12.32	4.35	30.44	53.34	164.85	0.36	–35.31
	ISO (10nM)	–69.96	17.17	11.67	22.13	34.58	111.41	0.44	–45.73
Updated (HZ_2)	CTR	–60.89	13.41	4.78	30.02	53.29	166.74	0.32	–39.30
	ISO (10nM)	–71.31	19.14	14.60	21.68	34.65	127.88	0.32	–49.34

Table SVI. Effects of 0.1 to 10 nM ISO on SAN pacing and model parameters.

Model		MDP (mV)	OS (mV)	dV/dt_{max} (V/s)	APD_{50} (ms)	APD_{90} (ms)	Pacing Frequency (HZ)	DDR (V/s)	TOP (mV)
CTR		–60.89	13.41	4.78	30.02	53.29	6.00	0.32	–39.30
ISO (nM)	0.1	-62.13	14.50	5.42	28.78	50.21	6.41	0.31	-43.28
	0.5	-65.53	17.03	7.49	26.40	44.21	7.14	0.32	-46.21
	1	-67.52	18.16	9.26	25.09	41.10	7.44	0.32	-47.36
	2	-69.27	18.91	11.35	23.72	38.28	7.63	0.32	-48.28
	10	-71.31	19.14	14.60	21.68	34.65	7.81	0.32	-49.34

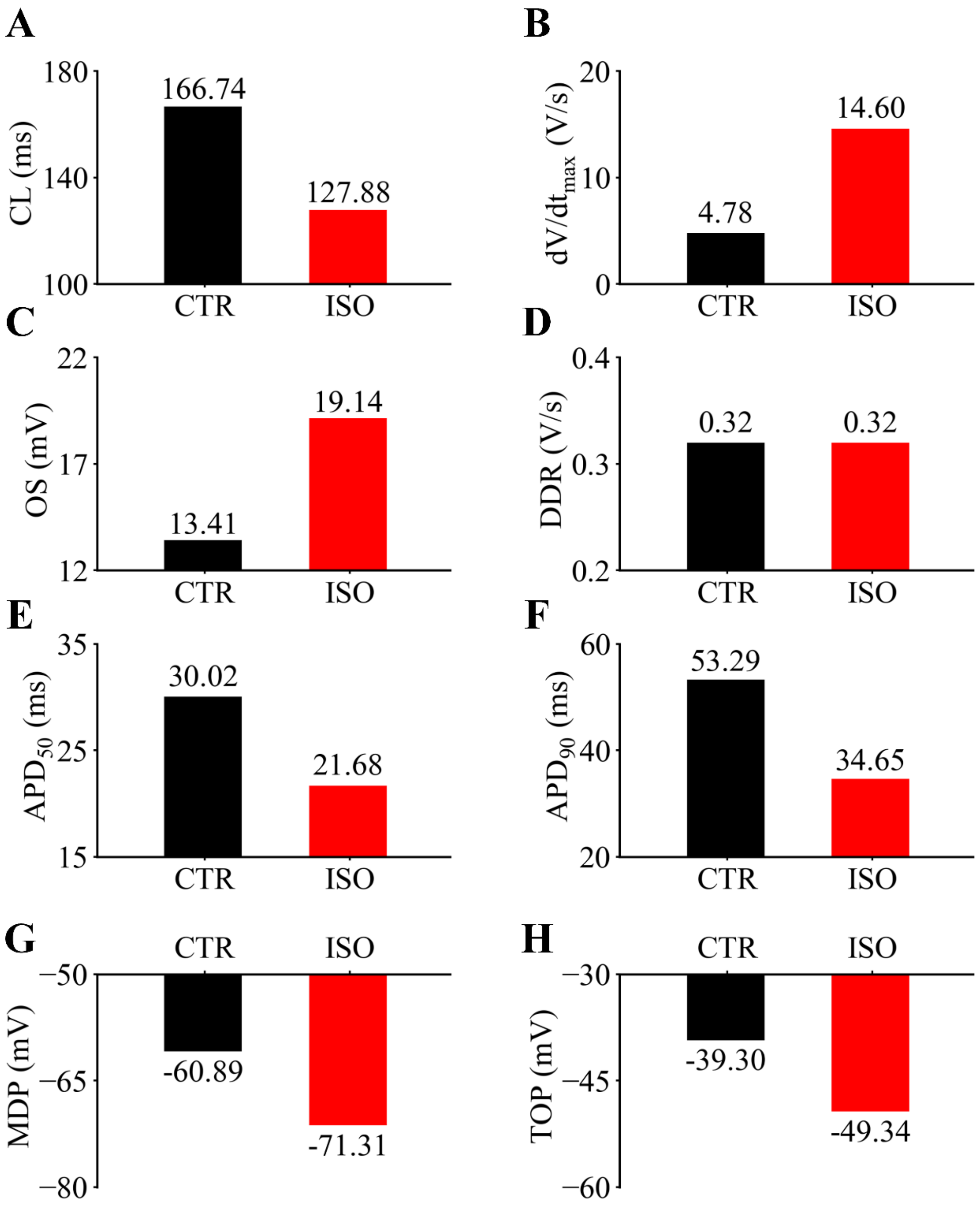

Figure S4. Effects of ISO (10 nM) on characteristics/biomarkers of pacemaker APs. (A) Cycle length (CL). (B) Maximum rate of phase 0 of the action potential (dV/dt_{max}). (C) Overshoot of AP (OS). (D) Maximum rate of the diastolic depolarization (DDR). (E) APD_{50}. (F) APD_{90}. (G) Maximum diastolic potential (MDP). (H) Threshold for firing a take-off potential (TOP).

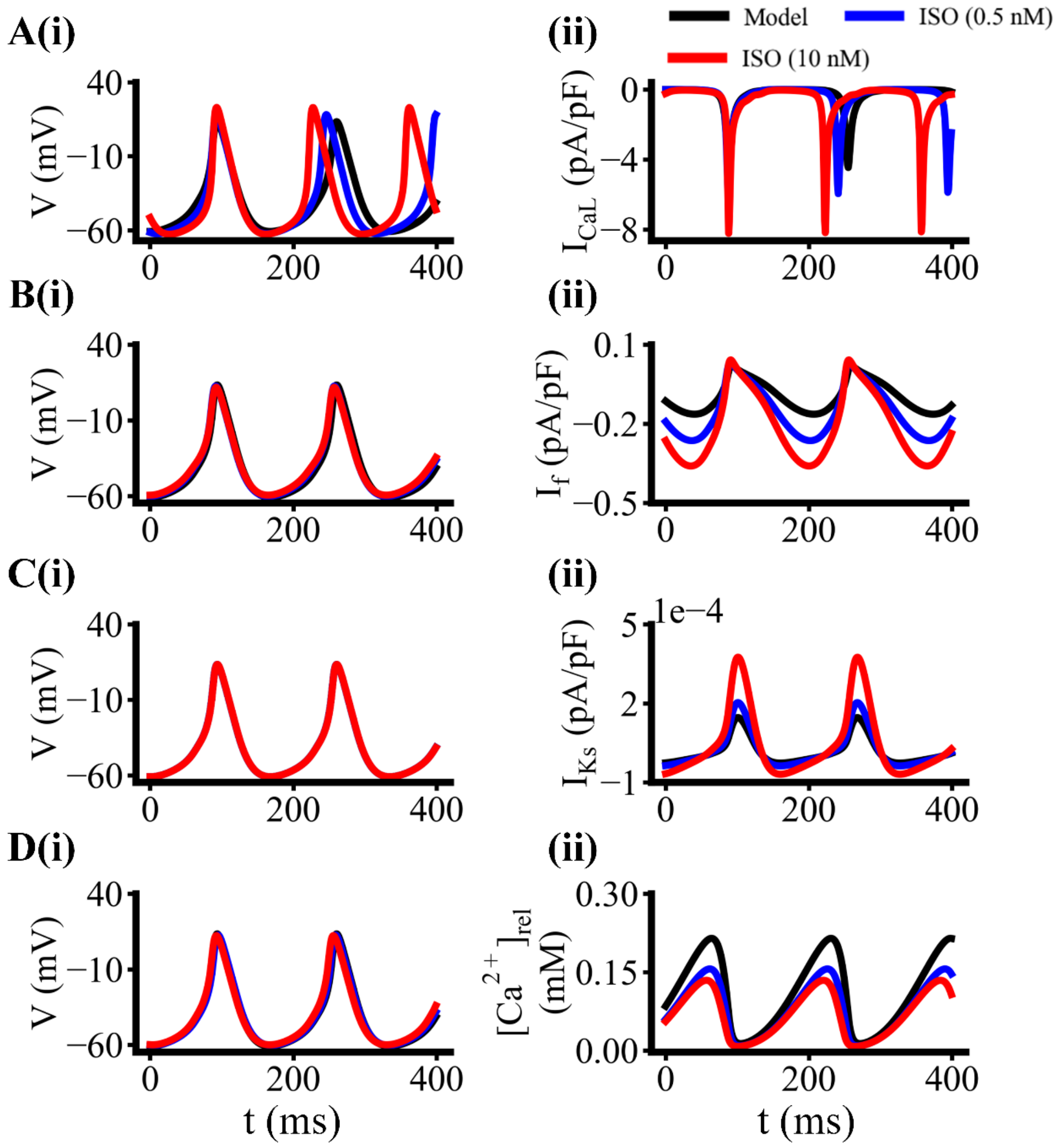

Figure S5. Simulated waveforms of spontaneous pacemaker potential (APs) (A(i)-D(i)) and underlying ion channel current or $[Ca^{2+}]_i$ in control condition and following the addition of ISO (0.5 or 10 nM). (A(ii) – D(ii)) when an individual ISO action of the designated ion channel/Ca^{2+} handling target is considered in the presence of two different ISO concentrations (control: black; 0.5 nM: blue; 10 nM: red). A: ISO action on I_{CaL} alone. B: ISO action on I_f alone is considered. C: ISO action on I_{Ks} alone is considered. D: ISO action on SR-Ca^{2+} release alone is considered.

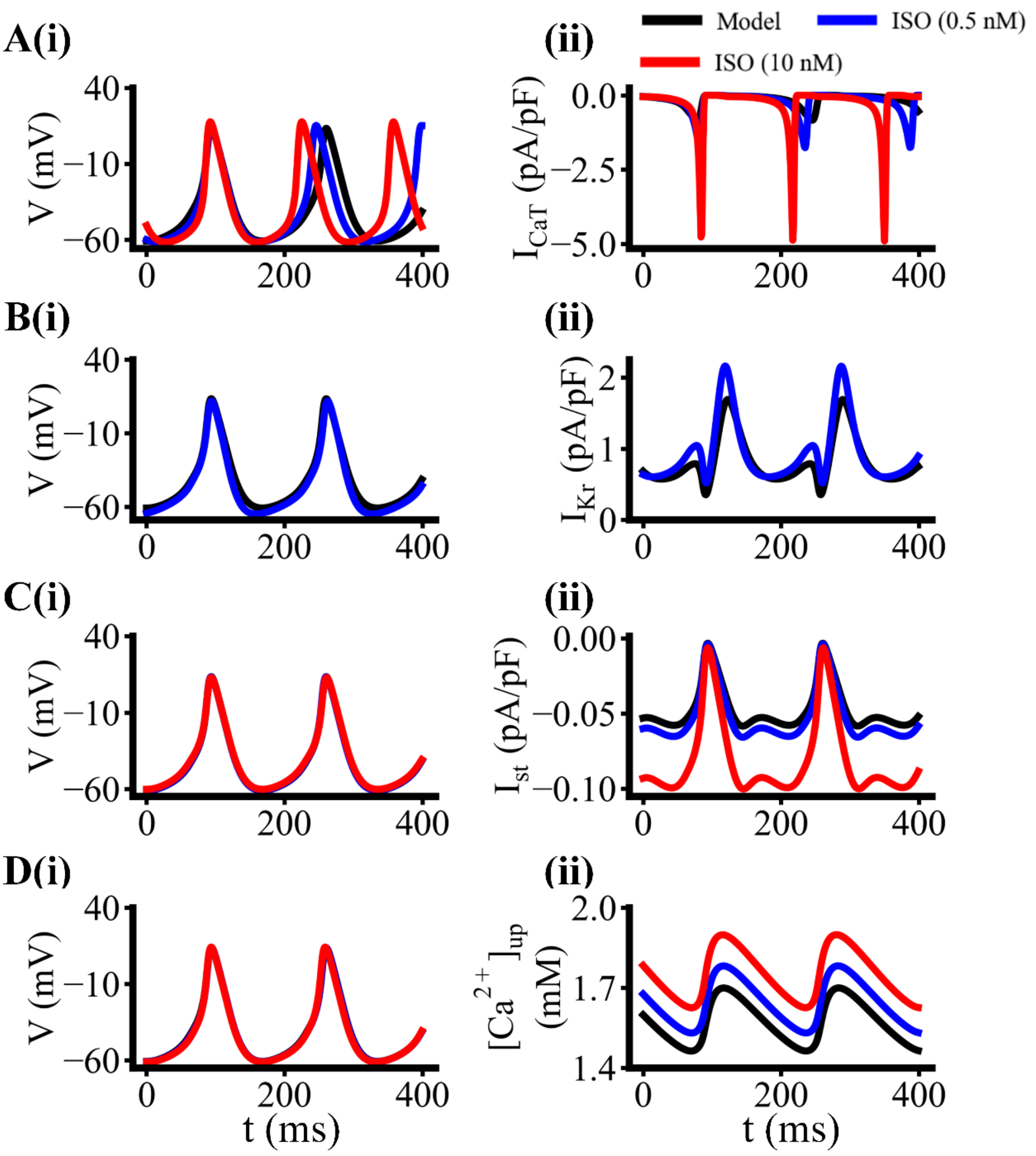

Figure S6. Simulated waveforms of spontaneous pacemaker potentials (APs) in control and following the addition of ISO (0.5 or 10 nM). (A(i)-D(i)) and underlying ion channel current or $[Ca^{2+}]_i$ (A(ii) – D(ii)) when an individual ISO action of the designated ion channel/Ca^{2+} handling target is considered in the presence of two different ISO concentrations (control: black; 0.5 nM: blue; 10 nM: red). A: ISO action on I_{CaT} alone. B: ISO action on I_{Kr} alone is considered. C: ISO action on I_{st} alone is considered. D: ISO action on SR-Ca^{2+} uptake alone is considered.

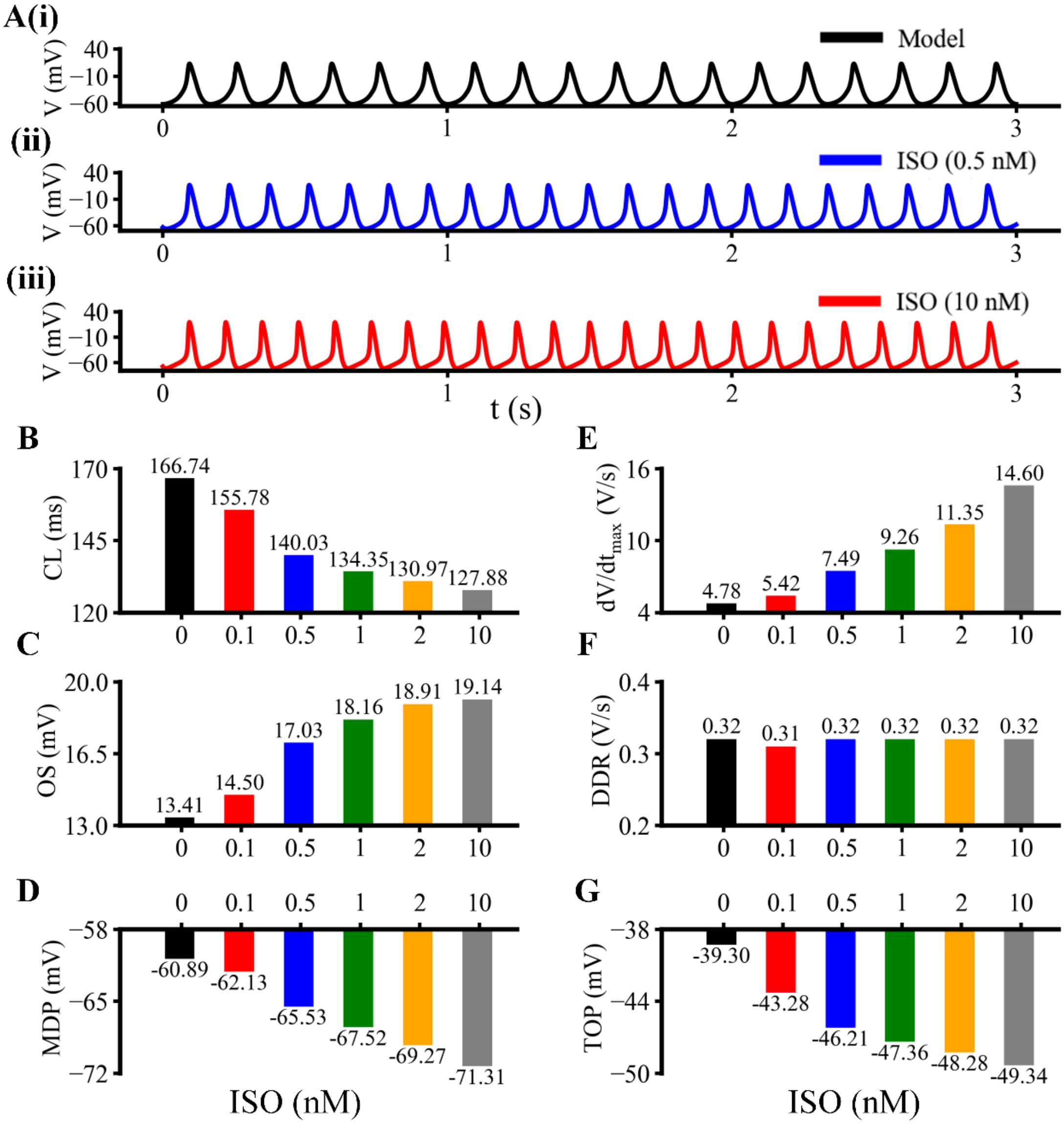

Figure S7. Effects of two selected concentrations of ISO on pacemaking and action potential waveform and corresponding biomarkers. (A(i)-A(iii)): APs for control (black), ISO 0.5 nM (blue) and ISO 10 nM (red). (B-G) Summary histograms illustrate the effects of different concentrations of ISO on CL (B), OS (C), MDP (D), dV/dt$_{max}$ (E), DDR (F) and TOP (G). All abbreviations are the same as those provided in legend of Figure S4.

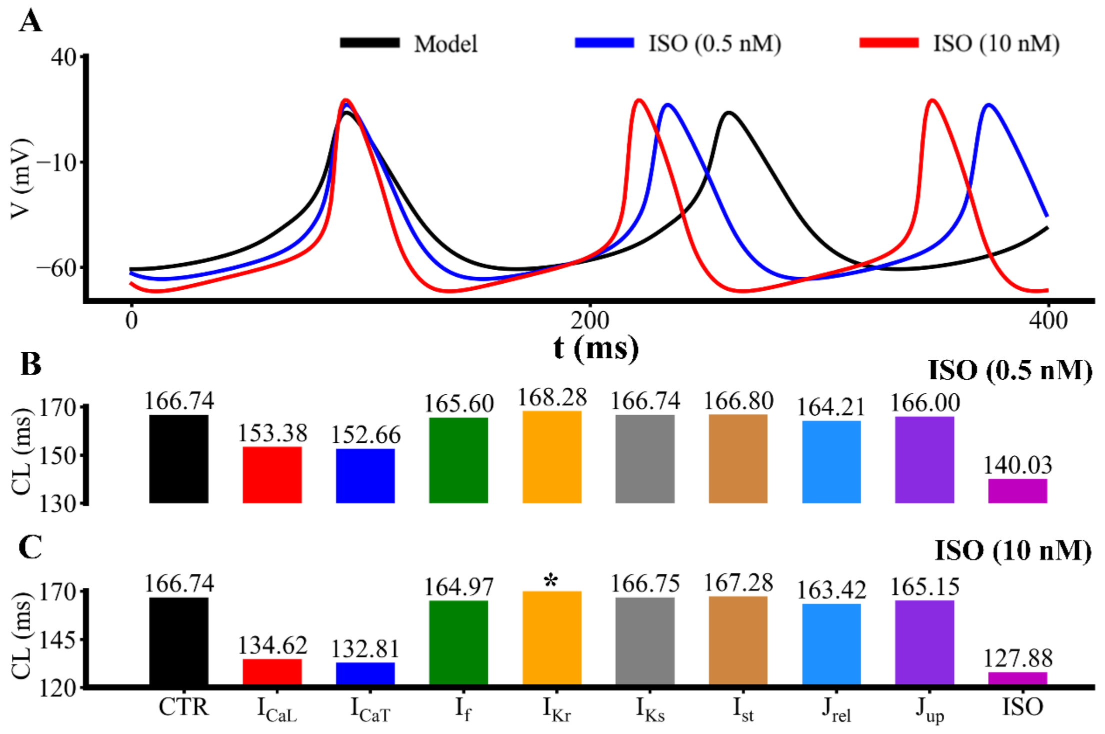

Figure S8. Illustrations of ISO-induced changes on individual ion channel parameters or the intracellular Ca^{2+} handling biomarker during spontaneous pacemaking and APs. A: Spontaneous APs in control, ISO (0.5 nM) and ISO (10 nM) conditions when all ISO-induced changes are integrated illustrated. B: Effects of individually "targeted" actions of ISO (0.5 nM) (I_{CaL}, I_{CaT}, I_f, I_{Kr}, I_{Ks}, I_{st}, J_{rel} (Ca^{2+} release flux from the JSR to the subspace), J_{up} (Ca^{2+} uptake flux from the myoplasm to the NSR) on CL.

C: Effects of individual action of ISO (10 nM) (including I_{CaL}, I_{CaT}, I_f, I_{Kr}, I_{Ks}, I_{st}, J_{rel} (Ca^{2+} release flux from the JSR to the subspace), J_{up} (Ca^{2+} uptake flux from the myoplasm to the NSR)) on CL.

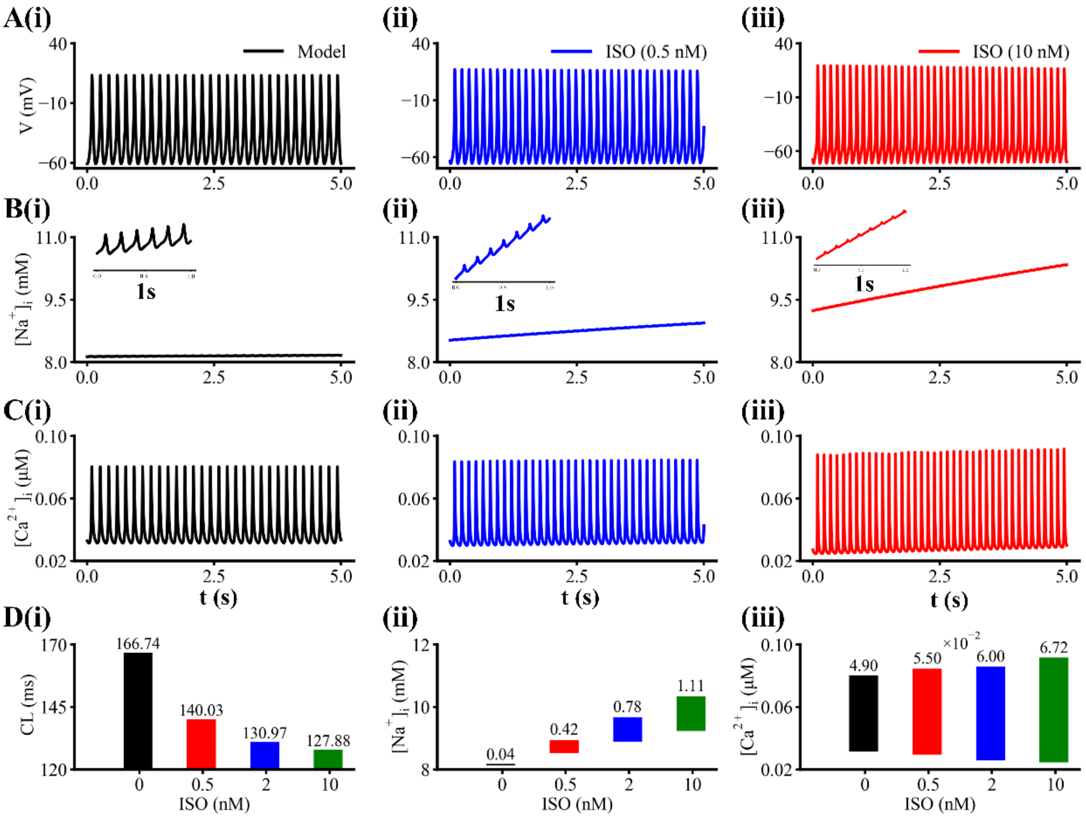

Figure S9. Effects of 2 selected ISO concentrations on AP waveforms, pacemaking and intracellular concentrations of Na^+ and Ca^{2+}.

A(i)-A(iii): APs at control, and in ISO (0.5 nM) and ISO (10 nM) conditions.

B(i)-B(iii): $[Na^+]_i$ at control, ISO (0.5 nM) and ISO (10 nM) conditions. Note that $[Na^+]_i$ remains relative stable, though a small drift (increase) seen in the enlarged scale (inset) during the long-term simulation. A similar drifting in $[Na^+]_i$ is also seen in ISO conditions.

C(i)-C(iii): $[Ca^{2+}]_i$ at control, ISO (0.5 nM) and ISO (10 nM) conditions. Transient changes in $[Ca^{2+}]_i$ arise from a stable diastolic baseline in control but diastolic $[Ca^{2+}]_i$ appears to increase somewhat in ISO conditions.

D(i)-D(iii): Summary histograms illustrating ISO dose-dependence of CL, minimal and maximal range of $[Na^+]_i$ and $[Ca^{2+}]_i$ at control, ISO (0.5 nM) and ISO (10 nM) conditions.
